# Using Drugs to Probe the Variability of Trans-Epithelial Airway Resistance

**DOI:** 10.1371/journal.pone.0149550

**Published:** 2016-02-29

**Authors:** Kendra Tosoni, Diane Cassidy, Barry Kerr, Stephen C. Land, Anil Mehta

**Affiliations:** 1 Division of Cardiovascular and Diabetes Medicine, University of Dundee, Medical Research Institute Ninewells Hospital and Medical School, Dundee, Scotland, United Kingdom; 2 School of Medicine, University of Dundee, Dundee, Scotland, United Kingdom; Central Michigan University School of Medicine, UNITED STATES

## Abstract

**Background:**

Precision medicine aims to combat the variability of the therapeutic response to a given medicine by delivering the right medicine to the right patient. However, the application of precision medicine is predicated on a prior quantitation of the variance of the reference range of normality. Airway pathophysiology provides a good example due to a very variable first line of defence against airborne assault. Humans differ in their susceptibility to inhaled pollutants and pathogens in part due to the magnitude of trans-epithelial resistance that determines the degree of epithelial penetration to the submucosal space. This initial ‘set-point’ may drive a sentinel event in airway disease pathogenesis. Epithelia differentiated *in vitro* from airway biopsies are commonly used to model trans-epithelial resistance but the ‘reference range of normality’ remains problematic. We investigated the range of electrophysiological characteristics of human airway epithelia grown at air-liquid interface *in vitro* from healthy volunteers focusing on the inter- and intra-subject variability both at baseline and after sequential exposure to drugs modulating ion transport.

**Methodology/Principal Findings:**

Brushed nasal airway epithelial cells were differentiated at air-liquid interface generating 137 pseudostratified ciliated epithelia from 18 donors. A positively-skewed baseline range exists for trans-epithelial resistance (Min/Max: 309/2963 Ω·cm^2^), trans-epithelial voltage (-62.3/-1.8 mV) and calculated equivalent current (-125.0/-3.2 μA/cm^2^; all non-normal, P<0.001). A minority of healthy humans manifest a dramatic amiloride sensitivity to voltage and trans-epithelial resistance that is further discriminated by prior modulation of cAMP-stimulated chloride transport.

**Conclusions/Significance:**

Healthy epithelia show log-order differences in their ion transport characteristics, likely reflective of their initial set-points of basal trans-epithelial resistance and sodium transport. Our data may guide the choice of the background set point in subjects with airway diseases and frame the reference range for the future delivery of precision airway medicine.

## Introduction

The ciliated airway epithelium is the first line of host defence against airborne assault [1]. One measure of the integrity of this epithelial barrier is the product of its electrical (ohmic) resistance and epithelial surface area, known as trans-epithelial electrical resistance (TER; Ω·cm^2^). The clinical relevance is that TER dysregulation may drive disease pathogenesis. For example, it has been proposed that chronicity in asthma may be cued by an initial genetic and or environmental propensity that lowers TER, facilitating epithelial penetration that subsequently drives irreversible airway remodelling [2]. The clinical importance of TER is further illustrated by recent data suggesting that cigarette smoke, inhaled pollutants or acid exposure due to gastro-oesophageal reflux can all dysregulate tight junctional proteins by signalling through ion channels and/or acid sensors [3–5]. Furthermore, in Cystic Fibrosis (CF), mutation of one apical channel, the CF transmembrane conductance regulator (CFTR), disturbs a regulatory (proteostasis) network that, *inter alia*, controls TER [6]. Additionally, understanding TER regulation may also be important for new therapies aimed at rare inherited airway diseases, where a better understanding of the means to lower TER might aid penetration of agents targeted to repairing innermost progenitor cells that renew damaged airways [7,8]. Here, we focus on TER in primary human nasal epithelial (HNE) cells grown at air-liquid interface (ALI) with the caveat that many different methods exist to culture such cells [9–11] but as yet, open standard operating procedures that define a “normal” range of values for TER are not available. Perhaps a better term might be reference range because ‘normal’ is not agreed. Moreover, transparency of reporting is not aided by the polar opposite opinions about the relevance of the magnitude of TER measured in a given *in vitro* experiment [12]. These decade old controversies are unresolved which prompted us to review the factors underlying the plasticity of TER values generated by human nasal epithelia reconstituted *in vitro* derived from apparently healthy volunteers. Hence, our aim was firstly, to publish our reference range for the distribution of baseline TER values across nasal turbinate derived ALI cultures; secondly, to determine the range of TER across multiple ALIs derived from a given volunteer and thirdly, to quantitate drug-induced changes in TER after sequential manipulation of sodium and chloride ion transport using two differently ordered protocols (chloride transport stimulation after cAMP elevation followed by sodium transport inhibition, or vice versa). The data suggest that baseline TER is not normally distributed with dichotomous responses to drugs targeting ion channels. We propose measures to normalise the wide range of TER both at baseline and characterise the differential response to drugs acting on ion transport proteins such as the epithelial sodium channel (ENaC) and CFTR.

## Materials and Methods

### Materials

Penicillin/Steptomycin (#15070), Bovine Collagen I (#A10644), HBSS (#14025) and DMEM high glucose (#41966) were from Invitrogen (Paisley, Scotland, UK). Primocin (#ant-pm-1) was from Invivogen (Toulouse, France). CellnTec media (#CnT-17) was from CalTag Medsystems (Buckingham, England, UK). PromoCell Airway Epithelial Growth media (#C21160) was from PromoCell (Heidelberg, Germany). Corning Costar Snapwells and Transwells (#3450 and #3407 respectively) were from VWR (Lutterworth, England, UK). Accutase (#A6964), all-trans retinoic acid (#R2625), forskolin (#F6886), CFTR inhibitor Inh172 (#C2992), amiloride (#A7410), benzamil (#B2417) and BSA (#A7906) were from Sigma-Aldrich (Poole, England, UK). Monoclonal antibody anti-acetylated alpha tubulin (# ab24610, 1:1000) was from Abcam (Cambridge, England, UK). Cell Signalling polyclonal anti-Z0-1 (# 07D12, 1:1000) was from NEB (Hitchin, England, UK). Alexa Fluor 488 donkey anti-mouse IgG (#A21202, 1:2000) and Alexa Flour 555 donkey anti-rabbit IgG (# A31572, 1:2000) were from Life Technologies (Paisley, Scotland, UK). Hydromount (#HS-106) was from National Diagnostics (Hessle, England, UK).

### Brush Biopsies

Nasal brushings from the inferior turbinate bones were performed on 18 healthy volunteers (12 male, 6 female, age range: 18–40 (mean 29) years) as previously described [13–15]. The brushing procedure was approved by East of Scotland Research Ethics Service (EoSRES) REC1 (REC reference 12/ES/0081 Protocol number 2011RC20), and informed written consent was obtained from all subjects (A4 documentation signed and retained).

### Cell culture

The cell culture protocol is based on a combination of different approaches [16–18] leading to our standard operating protocol (SOP); where reference is made to additional notes these may be found in the S1 File. After initial screening studies using different cell culture media (n>30 donors, data not shown) we identified two commercially available serum-free media that efficiently propagated cells on T25 collagen-coated dishes. Both media were used as per manufacturer’s recommendation and additionally supplemented with 2% Penicillin/Streptomycin and 100μg/ml Primocin. From this initial work, a culturing methodology was optimized. Brushings were all collected in phosphate buffered saline (PBS) at room temperature and centrifuged at 122×*g* for 4 min. The cell pellet was washed once with PBS and then the cells were seeded onto flasks that were collagen-coated (Bovine Collagen I- note-1) at 5 μg/cm^2^ in PBS for at least 1 hour at 37°C. Prior to cell seeding, the plate surface was washed twice with PBS. Typically, each pooled brushing (from six nasal scrapes, three per side) was sufficient to seed two T25 flasks (note-2 to 4). Cells were firstly seeded in CellnTec media, for 48h (adherent cells were designated P01) on collagen-coated T25 flasks, after which the supernatant containing the non-adherent cells was centrifuged (4 min at 122×*g*) and the cells were resuspended in the PromoCell Airway Epithelial Growth media, and seeded in T25 flasks for another 48h (attached cells then designated P03). After that time, the supernatant containing the non-adherent, and mostly spinning ciliated spheroids was transferred into a new flask and used for other experiments while fresh CellnTec media was added back to the original P03 T25s. The attached P01/P03 cells were grown until 80–90% confluent (typically 7 to 10 days post-seeding, note-5), and exposed to a cell detachment solution (Accutase, 1ml per T25), monitoring until all cells detached. Cells were then re-seeded on to collagen-coated Transwell or Snapwell inserts, depending on the nature of the subsequent experiments. In general, from two T25 plates (either P01 or P03), 12 Snapwells and 2 Transwells could be seeded (note-6). The cells were grown in CellnTec media submerged until confluence was reached (typically after 2–3 days) and then the medium was removed on the apical side to establish ALI. Pro-differentiation media, which consisted of 1:1 ratio of DMEM high glucose and PromoCell containing 1X concentration of PromoCell supplements plus 50nM all-trans retinoic acid, was added to the basolateral side. Medium was changed in the lower chamber of the inserts and the cells were washed apically with cell media every two days (note-7 and 8). Trans-Epithelial Electric Resistance (TEER) was assessed *in situ* every three days using a chopstick Epithelial Voltohmmeter (EVOM2, World Precision Instruments -WPI, Stevenage, England, UK) and inserts with TEER >400 Ω/insert (note 9) were used for Ussing Chamber experiments. Ciliogenesis was typically observed after ∼3 weeks at ALI with evidence of tight junction formation.

### Ussing Chamber

Snapwell supports with confluent and resistive cells, were mounted in an Ussing chamber and bathed both apically and basolaterally with Hank’s Balanced Salt Solution (HBSS, composition mM: 137.93 NaCl, 4.17 NaHCO_3_, 1.26 CaCl_2_, 0.49 MgCl_2_, 0.41 MgSO_4_, 5.33 KCl, 0.44 KH_2_PO_4_, 0.34 Na_2_HPO_4_, 5.56 D-Glucose), bubbled with 5%CO_2_/ 95%O_2_ at 37°C. Experiments were performed as described in [19]. Briefly, the epithelia were maintained under open-circuit conditions and the spontaneous trans-epithelial potential difference (V) was monitored (DVC-1000 Voltage/Current Clamp, WPI) and recorded (4 Hz) electronically (ADI Powerlab Interface and associated software; AD Instruments, Chalgrove, Oxfordshire, UK). Experiments were initiated once V had stabilized (20–30 min) then standard pulses of trans-epithelial current (20 sec, −10 μA·cm^−2^) were injected every 40 sec, leaving the epithelia to stabilize again (10–15 min) before adding any drug. The spontaneous voltage generated by the cells is reflective of the *in vivo* lumen-negative voltage when an electrode is placed on a nasal turbinate and connected in series with a voltmeter attached to another electrode in basolateral space. This universally observed negative deflection of the turbinate surface electrode in humans is thought to be due to positively charged sodium ions moving towards the blood inferred by the near collapse of that voltage when the sodium blocker amiloride is added to the perfusate bathing the turbinate, or in the Ussing chamber equivalent, when amiloride is added to the apical side of the cultured cells. We parameterised the role of sodium transport by cation substitution experiments in order to prove that the voltage at baseline was sensitive to sodium withdrawal (with N-methyl-D-glucamine, NMDG, S1 Fig). Next, we undertook pilot experiments showing that amiloride and the high affinity ENaC blocker benzamil at a 10-fold lower dose (1μM, 10 min) were electrophysiologically indistinguishable (data not shown). Thereafter amiloride was used as the most cost effective, widely used inhibitor thereby facilitating others to compare their protocols against our reference range. Additionally, two different concentrations of the CFTR inhibitor gave the same inhibition of forskolin-stimulated ion transport (data not shown).

### Drug addition regimes

We determined how TER, V and the ratio of V/TER [I_Eq_] changed under two drug regimes in which drugs where added sequentially, without any washout.

1. To raise cAMP, forskolin (FSK) was added both apically and basolaterally at 10μM for 15 min, followed by the CFTR blocker, CFTR_Inh172_ apically either at 8.0 or 4.0 μM for 15 min. Amiloride (AMI) was finally added apically at 10μM for 10 min.
2. *Vice versa*, such that amiloride was added first followed by forskolin and CFTR_Inh172_, conditions as above.

The important caveat to the ion transport data reported herein is that the results reflect symmetrical apical and basolateral sodium chloride (137.9 mM) as this eliminates differences across the paracellular space. For each ALI, V, TER and [I_Eq_] values (measured or calculated) during 4 min window out of the 10–15 min initial stable state were averaged and termed Baseline (BAS.). The same approach was applied for the values after amiloride and CFTR_Inh172_ addition. For the means with forskolin, the time window for averaging was increased to 6 min.

Additional information relating to brush biopsies, cell culture (SOP and immunofluorescence) and Trans-epithelial resistance calculation can be found in the S1 File.

### Statistics

Analysis was performed with GraphPad Prism 6.0 (GraphPad Software Inc., La Jolla, CA, USA) and results expressed as means ± errors as specified in Fig legends. Data were compared using unpaired Student’s *t*-test or one-way ANOVA. Where tests of normality (Shapiro-Wilk) failed, data were analysed using non-parametric tests (Mann-Whitney *U* test and Kruskal-Wallis ANOVA + Dunn’s multiple comparison test). Groups of data were considered to be significantly different if P <0.05.

## Results

**Fig 1:**
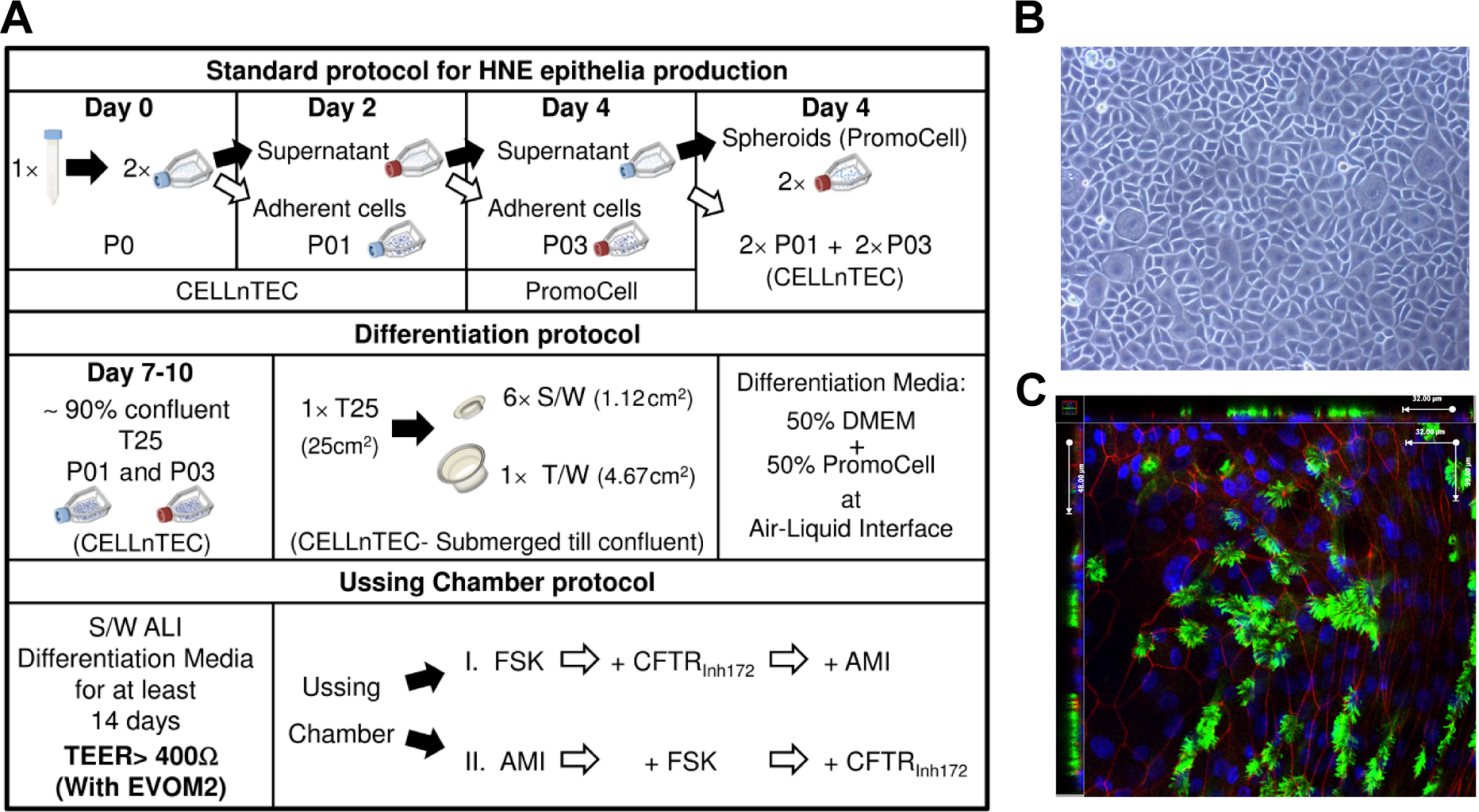
Human nasal epithelia *in vitro* reconstitution. (A) Schematic representation of cell culture protocol for expansion and differentiation of HNEs for Ussing Chamber experiments. (B) Typical HNE monolayer obtained after 7–10 days in culture on collagen-coated flasks. (C) Immunofluorescence of ALI differentiated epithelia: representative acetylated tubulin (green) and ZO-1 (red) with DAPI counterstain (blue) performed after Ussing chamber experiment; 3D reconstruction from z-stack in adjacent left and top panel.

Our final standard operating protocol (Fig 1A, top panel, P01 and P03 submerged ‘source’ cultures, Fig 1B) for the differentiation of human nasal epithelia was applied to 18 healthy donor biopsies. This SOP for differentiation at ALI generated ciliated HNE cultures, with spontaneous apical negative voltage (resistive after 15–20 days in culture) in >98% of brushings. A typical fully differentiated epithelium is shown in Fig 1C demonstrating both cilia and tight junction formation.

### Baseline electrophysiological values

**Fig 2:**
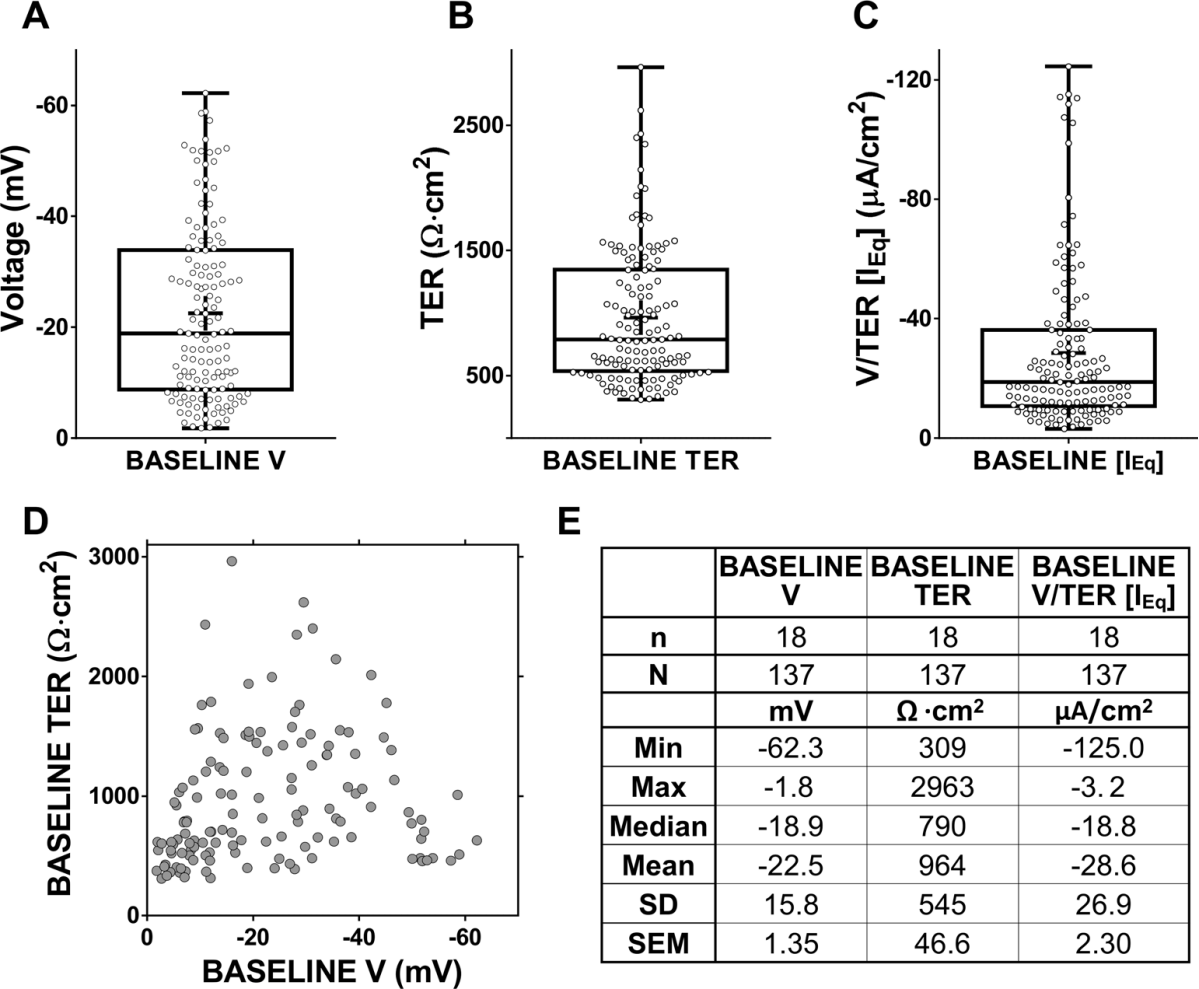
Baseline electrophysiological values from Ussing chamber experiments. (A-C). Box plots showing baseline electrophysiological values. Minimum, maximum (whiskers) and 25^th^/75^th^ percentile, median (box line) and mean (cross) are highlighted. (D) Plot of initial V against TER. (E) Table of basic statistical analysis of the data shown in A-C.

When ALI cultures developed resistance, each was mounted in an Ussing chamber with symmetrical solutions bathing apical and basolateral surfaces. Fig 2 shows the range of transport parameters at baseline from 137 ALI cultures (n = 18 volunteers). The variability of V, calculated resistance (TER) and equivalent current (V/TER[I_Eq_]) values is shown in Fig 2A, 2B and 2C respectively (summary in panel E).

These data also show no relationship between initial voltage (V) and calculated TER (Fig 2D). The baseline TER values were highly scattered with a ∼9-fold difference between the lowest and highest resistances (compare Min and Max Fig 2E), with most TER values clustered in the 500–800 Ω·cm^2^ range as reflected in the ‘Christmas tree’ shape of the distributions in Fig 2B (see also frequency distribution graphs in S2A Fig showing that nearly 60% of ALIs have TER between 300 and 900 Ω·cm^2^, 25% of the total in the range 500–700). Statistical analysis confirmed the non-normal distribution for all the parameters (P<0.001). This asymmetry at an ALI level (which persisted after logarithmic transformation of voltage and TER but not calculated current, data not shown) resulted from very high values in a minority of ALIs consistent with the idea that these have electrical properties that differ from the majority.

**Fig 3:**
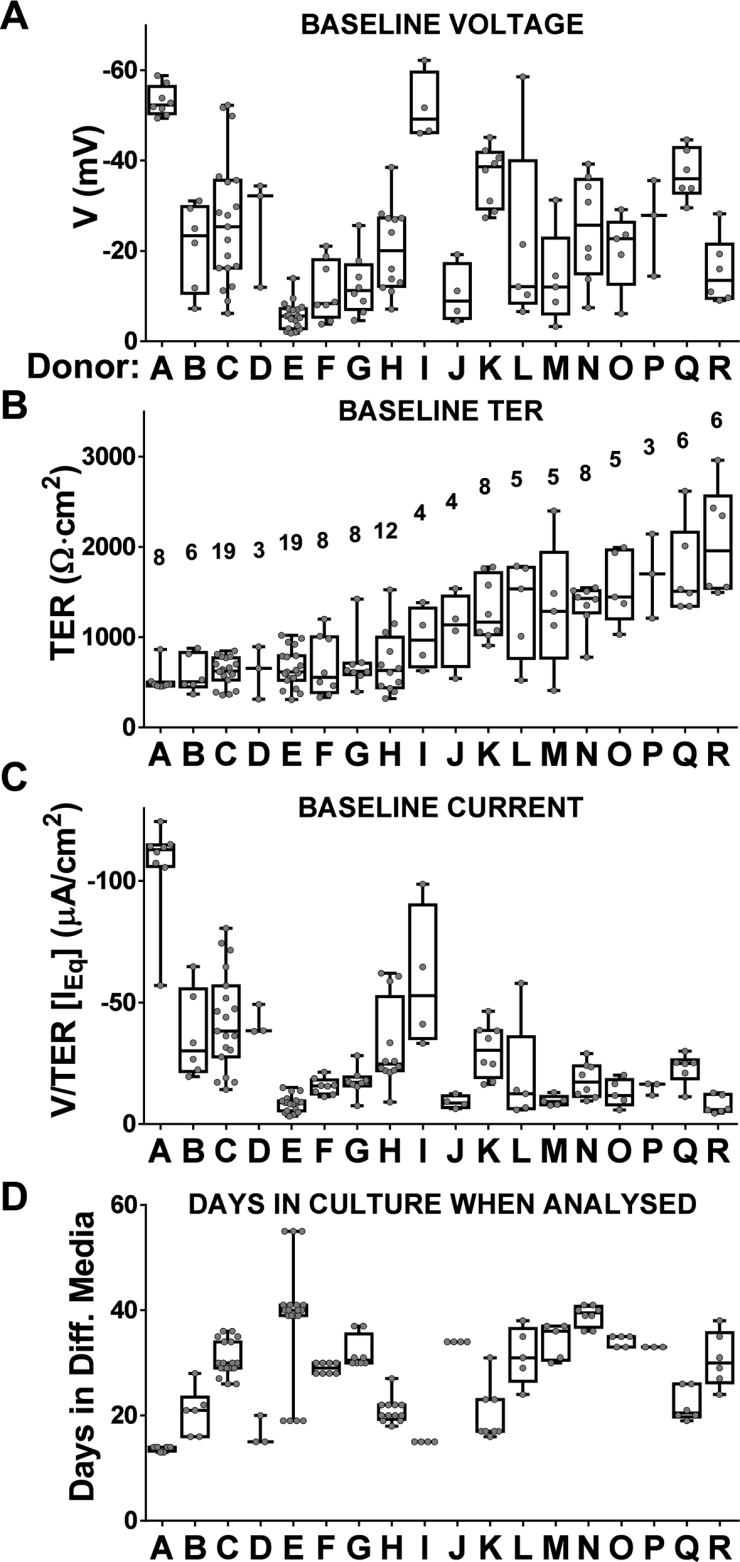
Electrophysiological values on an individual donor basis (A-R). Box plots per donor highlighting minimum, maximum (whiskers) and 25^th^/75^th^ percentile and median (box). Donors arranged in ascending order ranked by mean values of TER in (B) with number of ALIs per donor above the boxes. These are compared with either (A) voltage, (C) [I_eq_] and (D) time at ALI.

This finding that log transformation failed to normalise TER and voltage values prompted re-clustering at a volunteer level to test the hypothesis that high values were a characteristic of a given donor’s cells. First, we ranked the TER at baseline by donors mean values in ascending order (abscissa Fig 3B) and cross compared the spread of voltage and equivalent current (respectively Fig 3A and 3C).

As shown in Fig 2D, it was not possible given a starting voltage to predict the corresponding TER. Interestingly only a minority of ALIs from a single individual had a range of voltage values that spanned the extremes of the population distribution (e.g. donor C, H, L and N in Fig 3A). Fig 3D shows the length of time in culture at ALI in differentiation media to determine whether this could be used to predict the spread of the baseline values shown above.

As shown, there is no discernible relationship and the intra-donor variability cannot therefore be explained by the different length of time in which the cultures have been grown prior to Ussing Chamber analysis. Our data suggest that some individual donors vary significantly in their baseline TER (Fig 3B). In summary the non-normal distribution of the TER values is best explained by the observation that there are a greater number of individuals than expected at the extremes of the distribution. This means there are more individuals with a very low or high TER compare to a normal distribution as shown in S2 Fig.

### Drugs as probes to study TER plasticity

**Fig 4:**
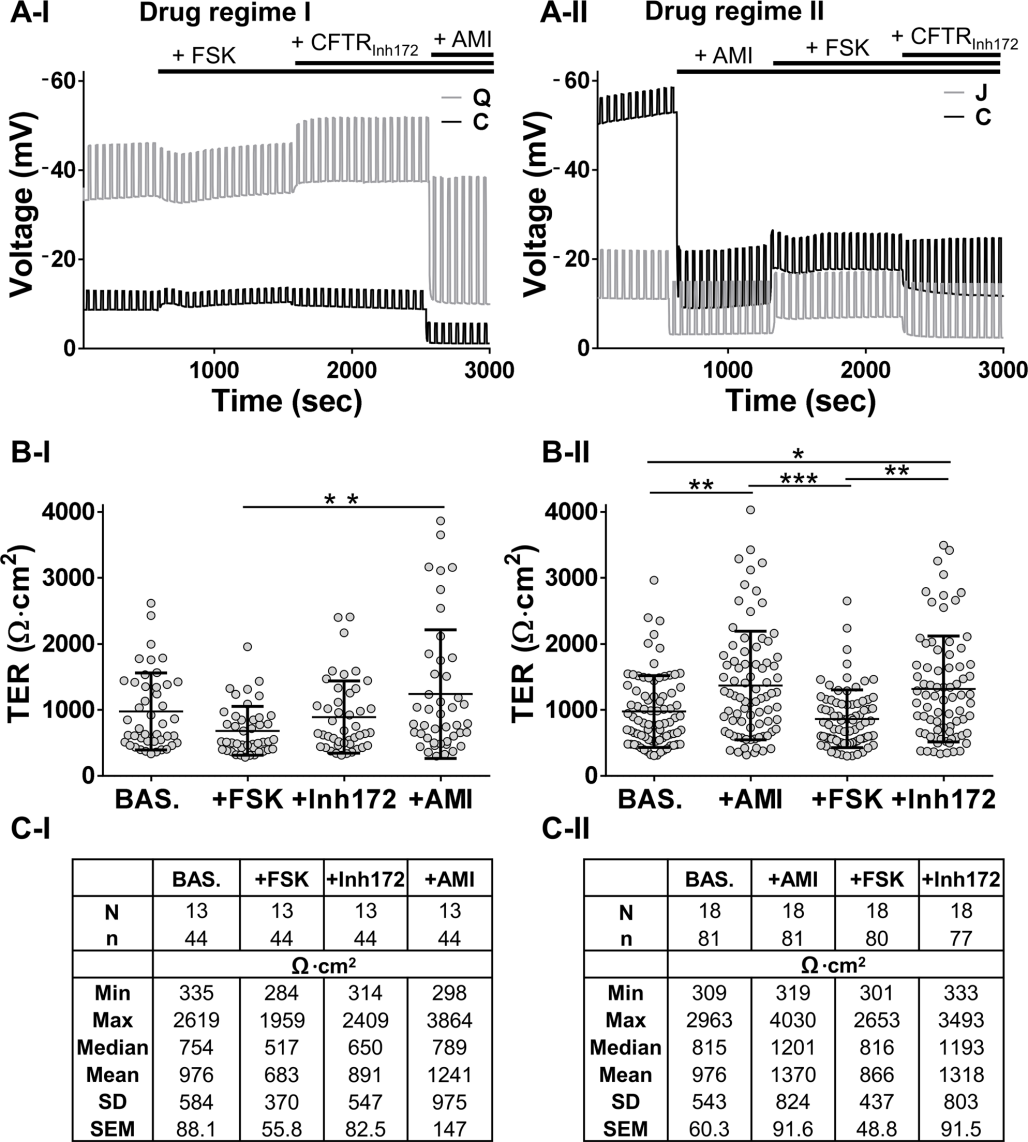
Ussing chamber ion transport analysis. (A-I, A-II) Two different drug regimes (I and II) were used and typical representative traces from two donors each are shown. (B-I, C-I) TER values obtained applying drug regime I with their Table of basic statistical analysis. Similarly (B-II, C-II) show the data obtained applying drug regime II. Error bars: mean ± SD; Kruskal-Wallis ANOVA + Dunn’s multiple comparison test: *P<0.05; **P<0.01, ***P<0.001.

Next, we investigated how TER values for a given ALI change in response to the sequential addition of drugs that elevate cyclic AMP (forskolin, FSK), inhibit sodium transport (amiloride, AMI), or inhibit CFTR (inhibitor CFTR_Inh172_). The rationale for the use of these drugs is described in the methods. We used two different orders of sequential addition of drugs (Fig 1A lower panel), as exemplified by the typical experimental traces in Fig 4A-I and 4A-II that also are reflective of the wide range of baseline voltages shown in Fig 2 (see also S3A Fig and Table A in S1 File for volunteers Q, C, J).

The sequential drug regime I was applied to 44 ALI cultures from 13 donors (Fig 4B-I and 4C-I) while drug regime II was tested using 81 ALI cultures from 18 donors (Fig 4B-II and 4C-II). The baseline TER values were similarly highly scattered (Min and Max Fig 4C-I and 4C-II). With drug regime I, ENaC was inhibited after pharmacological opening and closure of CFTR. Forskolin decreased TER by ~30% with respect to baseline, this effect being mostly counteracted by CFTR_Inh172_ (increase up to ~90% of BAS.). Subsequent amiloride addition raised TER by ~30% respect to baseline, also revealing a minority of ALI cultures whose resistance rose into the >3000 Ω·cm^2^ (+AMI in Fig 4B-I also S2 Fig, panel B-I for changes in relative frequency distribution of values). Variability of response was reflected in the 13-fold difference between the Min and Max values (+AMI, Fig 4C-I). This rise in a minority of ALIs is an amiloride-driven effect and not an artefact of the regimen since it recurred when regime II was applied (no prior chloride transport modulation, Fig 4B-II and 4C-II).

Upon amiloride addition TER increased by ~40% with respect to baseline; again very high resistances were observed in a minority of ALIs (see also S2 Fig, panel B-II). Forskolin decreased TER to ~90% of BAS., while CFTR_Inh172_ increased TER to ~35% above baseline. There was a considerable rise in the median value in the presence amiloride alone (from 815 to 1201), which was not observed in drug regime I when amiloride was added after forskolin and CFTR_Inh172_. This suggests that amiloride exposure after CFTR modulation was acting on the epithelium differently with respect to TER when compared to amiloride administered alone. Non-parametric statistical analysis demonstrated no significant difference between groups except +FSK vs +AMI in drug regime I. Contrastingly, drug regime II showed no significant difference between BAS. and +FSK, or between +AMI and +CFTR_Inh172_. See also S4 Fig (along with Tables E and F in S1 File, for mean data values) that shows how TER changes for all of the ALIs after the addition of drugs and also for the individual donor responses suggesting that certain individuals respond differently to drug administration. This variability prompted a deeper analysis.

### Initial baseline vs rolling baseline approach to data analysis

**Fig 5:**
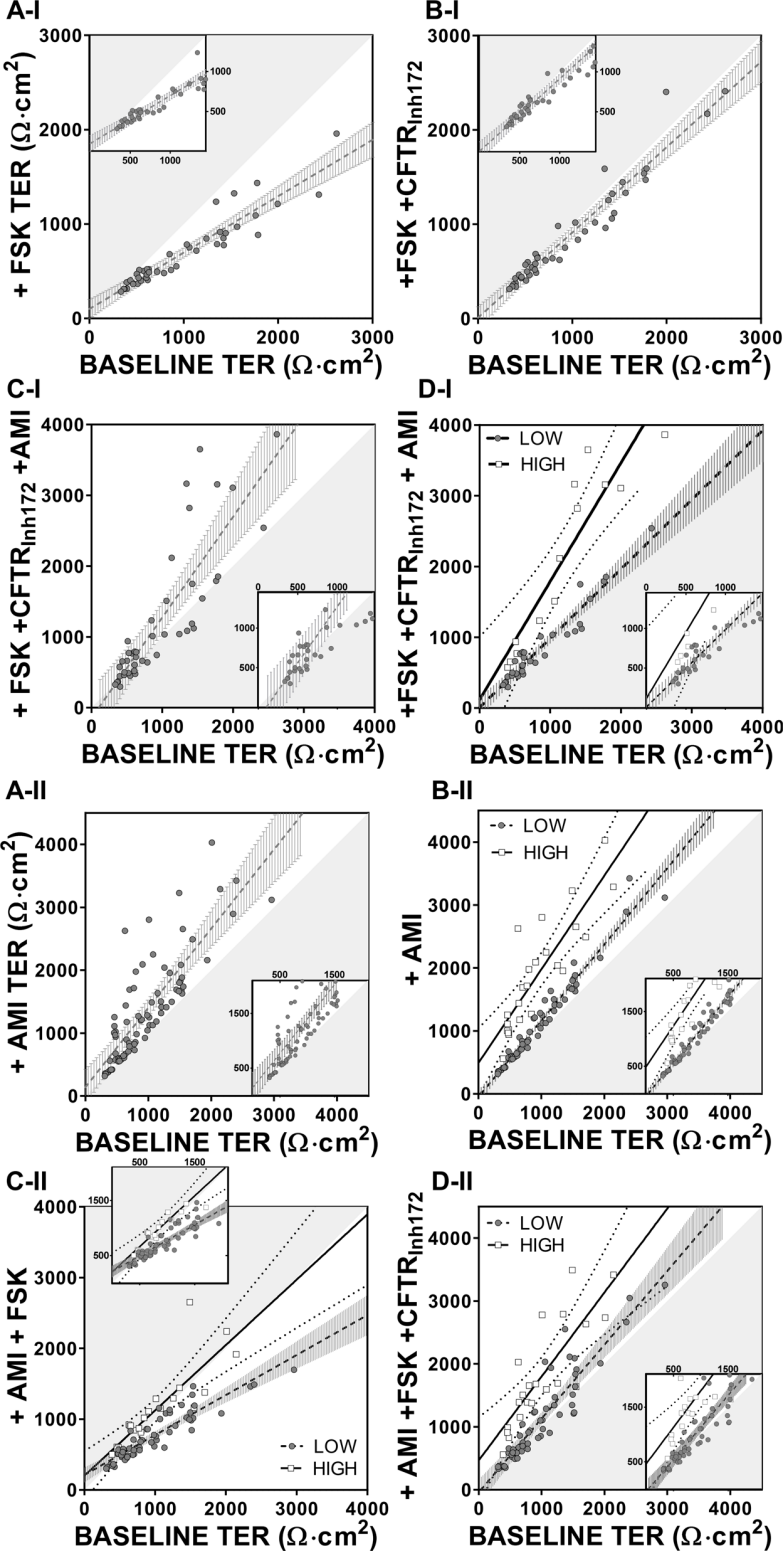
Drug induced effect on TER with respect to the initial baseline. TER values at baseline are plotted against values obtained after the cumulative addition of the different drugs. The hypotenuse of the shaded triangular area represents the line of identity, while magnifications are shown in the box inserts. (A-I) The initial baseline TER values (closed grey circles) are plotted against the values obtained after addition of FSK (n = 44), (B-I) FSK+CFTR_Inh172_ (n = 44) and (C-I) FSK+CFTR_Inh172_+AMI (n = 44); line of regression is shown as a dashed line with 99% CI (grey vertical bars). In D-I same data as in C-I showing two distinct populations of amiloride responders designated as LOW (closed grey circles-black dashed regression line +99% CI vertical bars, n = 31) and HIGH (open squares, black regression line +99% CI dotted line, n = 13). (A-II) The initial baseline TER values (closed grey circles) are plotted against the values obtained after addition of amiloride; line of regression is shown as a dashed line with 99% CI (grey vertical bars). In B-II same data as in A-II showing two distinct populations designated as LOW (n = 57) and HIGH (n = 24) amiloride responders. Changes in TER with cumulative drug addition of (C-II) AMI+FSK (LOW n = 56, HIGH n = 24) and (D-II) AMI+FSK+CFTR_Inh172_ (LOW n = 56, HIGH n = 21). Additional regression and statistical analysis data are shown in Table B in S1 File.

These analytical complexities prompted us to re-examine drug induced effects on TER using a different approach reflective of the starting value of TER. First, we determined whether the baseline TER was predictive of the degree of change upon drug addition (Fig 5).

Initially, we plotted the drug-induced TER values against a common starting baseline TER, for each regime (Fig 5A–5D, Drug regime I and II). The hypotenuse of each shaded triangular area in each panel of Fig 5 is the line of identity between the TER response to a drug (ordinate) and the baseline (abscissa), the latter being the start value of TER. It can be seen that in drug regime I, forskolin induced a fall in TER irrespective of the magnitude of the baseline TER (Fig 5A-I, inset). Interestingly CFTR_Inh172_ restored the TER back to the line of identity suggesting that the forskolin effect in lowering TER was largely driven by CFTR activation. Under these conditions, AMI induced a rise in TER to above the line of identity, but only for a minority of ALIs (Fig 5C-I) suggesting that there may exist two populations of amiloride-insensitive (data near the shaded area) and amiloride-sensitive ALIs (data lying outside the confidence intervals in the main Fig 5C). This is further exemplified in Fig 5D-I, where the low amiloride responders (LOW, closed grey circles) have been separated from the high responders (HIGH, open squares). The cut off to determine our division of this population into two groups was based on whether the fold increase of TER was above or below the slope value in Fig 5C-I. To test the validity of this arbitrary choice, we repeated this analysis with drug regime II.

On this occasion however, amiloride induced a rise in TER for all ALIs, but of different magnitude enabling the discernment of two groups of responders, LOW and HIGH, grouped by their fold rise in TER i.e. an increase being above or below the mean of the population (Fig 5A-II and 5B-II). Once again forskolin drove the TER below the line of identity (but only for LOW responders, see regressions in Table B in S1 File) and the two groups of LOW and HIGH responders remained quite distinct even when the CFTR_Inh172_ was superimposed (Fig 5C-II and 5D-II). This analysis shows that the changes in TER after the addition of drugs are independent of the baseline magnitude and the major changes that occur upon amiloride addition are specific to this compound, but partially affected by the order of its administration with respect to modulation of anion transport.

**Fig 6:**
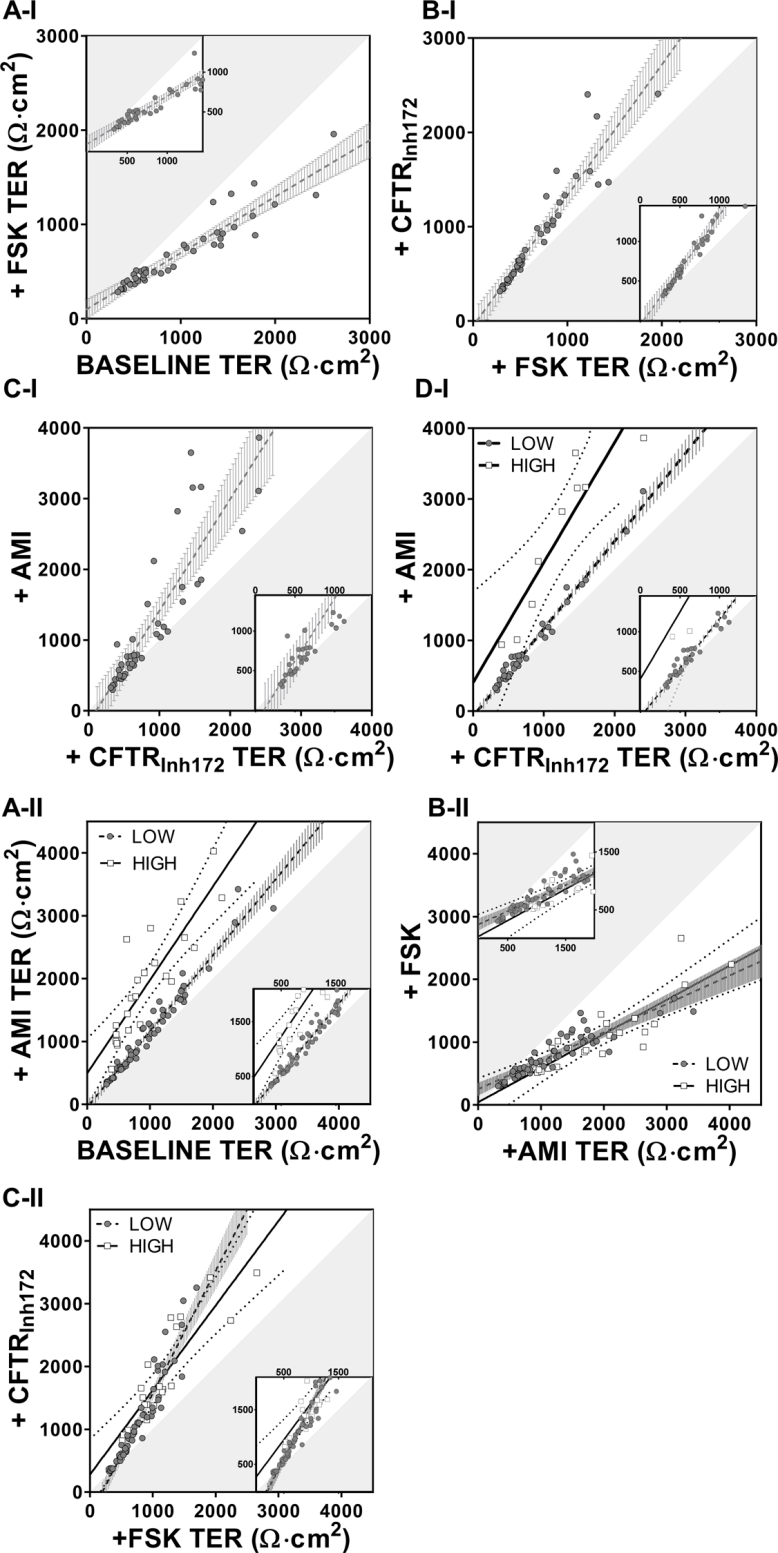
Drug induced effect on TER with respect to the rolling baseline. TER values at each stage of the drug regime chronologically are considered as baseline. The hypotenuse of the shaded triangular area represents the line of identity while magnification is shown in the box inserts. (A-I) The initial baseline TER values (closed grey circles) are plotted against the values obtained after addition of forskolin (n = 44). (B-I) TER values +FSK are plotted against values after addition of CFTR_Inh172_ (n = 44). (C-I) Values of TER with CFTR_Inh172_ plotted against values +AMI (n = 44); line of regression: dashed line +99% CI (grey vertical bars). In D-I same data as in C-I showing two distinct populations of amiloride responders designated as LOW (closed grey circles-black dashed regression line +99% CI vertical bars, n = 35) and HIGH (open squares, black regression line +99% CI dotted line, n = 9). (A-II) The initial baseline TER values are plotted against the values obtained after addition of amiloride showing two distinct populations designated as LOW (n = 57) and HIGH (n = 24) amiloride responders. (B-II) Amiloride values plotted against values +FSK (LOW n = 56, HIGH n = 24). (C-II) Values after addition of forskolin plotted against values +CFTR_Inh172_ (LOW n = 56, HIGH n = 21). Additional regression and statistical analysis data are shown in Table C in S1 File.

Next, we re-plotted the data but now assuming that the TER value in the presence of a given drug generated a new drug-induced baseline with each addition (Fig 6).

Using this rolling baseline approach, in Fig 6B-I, CFTR_Inh172_ doubles the slope (compare with panel A-I). Subsequent addition of amiloride increases the slope yet further (Fig 6C-I), and the two groups of LOW and HIGH responders are now discrete (Fig 6D-I). In regime II, where amiloride is added first, differences between the LOW and HIGH groups were more difficult to discern when more than one drug was present showing differences in the effect of CFTR_Inh172_ but not forskolin. (Fig 6B-II and 6C-II, see slope t-test, Table C in S1 File). S5 Fig shows the data redrawn for ease of comparison. First, the baseline resistance is used as a common reference point (S5 Fig, panels A-I and A-II). Alternatively, each drug-induced resistance value is used as a rolling reference point where the focus is on the individual effect of a given drug (S5 Fig, panels B-I and B-II).

**Fig 7:**
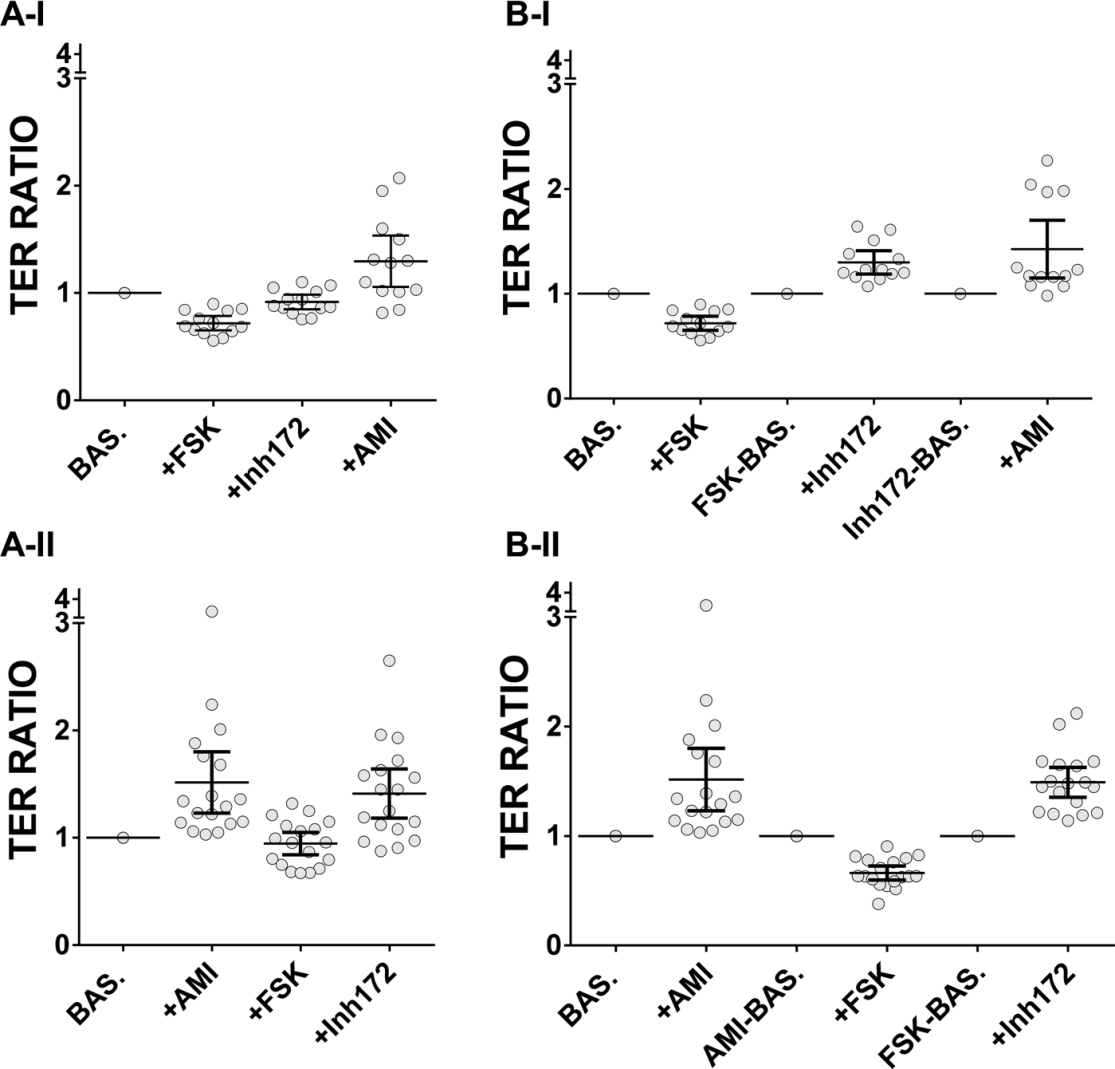
Donor-level analysis of TER ratio change upon drug addition. (A-I, A-II) Donors averaged ratios of TER with drug regime I (N = 13) and II (N = 18) respectively, applying the initial baseline approach highlighting the cumulative effect of the drugs. (B-I, B-II) Same data analysed applying the rolling baseline approach to the TER mean values of the individual donors. Error bars: mean ± 95% CI.

The response patterns are qualitatively different but in each case outliers become apparent as certain drugs are added, with each inhibitor of ion transport showing the greatest outlier generating effect (see also S6 Fig for a quantitative analysis of the range of outliers). Earlier, we had found that individual donors’ data were reflective of ALI data as a whole. Hence, we re-analysed the data at a volunteer level. Fig 7B-I shows that upon amiloride addition two distinct groups of volunteers are revealed in regime I using the rolling baseline approach, whose existence is implied but not clearly discriminated using the other drug regime (+AMI Fig 7A-II and 7B-II). For completeness we also show the mean responses when the reference is set to the initial baseline TER (Fig 7A-I and 7A-II).

**Fig 8:**
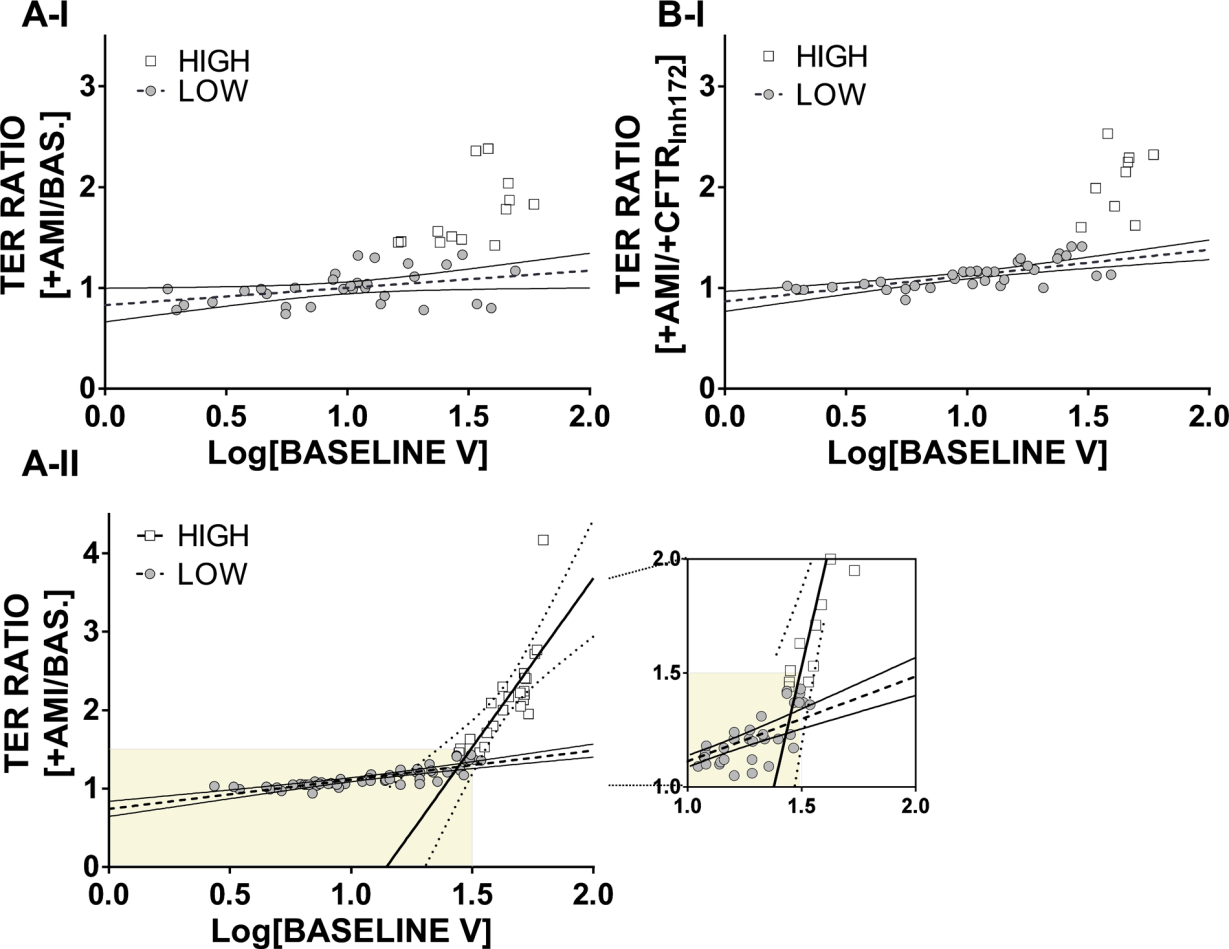
Relationship between baseline voltage and response of TER after amiloride addition. Logarithm of baseline V plotted against ratio of TER upon amiloride addition in drug regime I, applying the initial baseline (A-I), or the rolling baseline approach (B-I). In drug regime II (A-II), grouping produced a significant difference between the two groups (LOW, n = 57; HIGH, n = 24). For both drug regimes, grouping was performed as in Figs 5 and 6. Additional regression and statistical analysis data are shown in Table D, S1 File.

Our initial findings were that there was no relationship between baseline voltage and baseline TER. However, when the initial voltage is plotted for a given ALI against its TER ratio response to amiloride addition, we now observe a curvilinear relationship between the two parameters (S7 Fig). Alternatively, plotting the logarithm of the initial voltage, two distinct populations emerge, especially in drug regime II; and also in drug regime I but only when the TER ratio is recalculated as +AMI/+CFTR_Inh172_ (rolling approach, S7 Fig, panel D-I). Applying the grouping as in Figs 5 and 6, the two populations of LOW and HIGH responders remain distinct (Fig 8), but are more clear with drug regime II (Fig 8A-II, Table D in S1 File).

These graphs show that the HIGH amiloride responders have higher baseline voltages. Importantly, from the comparison of the groups obtained with either of the drug regimes and the different analytical approaches (Figs 5 and 6), we observed that the outliers in the presence of amiloride (HIGH responders, for example Fig 7B-I and 7A-II), belong to the same donors irrespective of whether amiloride is added first or last (compare also donors with ratios above 1.5 for +AMI in S6 Fig, panel B-I and A-II). This suggests that healthy volunteers demonstrate significantly different changes in TER in response to drugs such as amiloride that fall into dichotomous groups after drug challenge.

## Discussion

Many research groups use cultured nasal brushings to model airway function [9,20], albeit with differences in opinion on the relevance of such biopsies [12,21], which is not surprising given the many different protocols for their cell culture together with different methodologies to assay bioelectrical properties [22]. Our results are comparable to the literature for either nasal [16,23] or tracheal/bronchial [11,24] epithelial cells, and those from commercial sources [25]. Our observed spread of values, i.e. intra-donor variability between ALIs from a given biopsy, is most likely caused by differences in the seeding composition of progenitors (Fig 1A), since this variability cannot be explained by differences in the length of time in culture as shown in Fig 3D. These causes are beyond the scope of the current paper but have been speculated on recently [26]. Such TER variability poses a severe challenge for the very idea of normal controls which in turn becomes a critical issue in the interpretation of disease findings and especially when any therapy has to be personalised, also known as precision medicine [27]. First, we find a large range, irrespective of whether the mean or median values from a given volunteer’s aggregated ALI data are studied. Second, our transport data are not normally distributed with voltages over a >30-fold range (-62.3/-1.8 mV), with an equivalent >9-fold range of values for TER (from 309 to 2963 Ω·cm^2^) coupled to a minority of ALIs that have drug responses which differ markedly from the rest. Third, this variability is likely not an *in vitro* artefact because such a wide range is also found *in vivo* when nasal potential difference is measured in apparently healthy volunteers [28,29] which is of clinical importance given that this test has been proposed as a discriminant between disease and health [30]. Fourth, the volunteers who have a higher response to amiloride are the same subjects irrespective of the order of drug addition, suggesting that the degree of amiloride sensitivity is an intrinsic property of an individual’s epithelium. Fifth, a high amiloride response, is not predictive of an equivalently high response to forskolin plus CFTR_Inh172_ (i.e. donor A vs R in S6 Fig). Sixth, there is no clear relationship between baseline voltage and resistance (Fig 2D). This is not unexpected given the complexity of the regulation of sodium transport across the airway epithelium [31] as the dominant driver of baseline voltage coupled to the equivalent complexity of the dynamic regulation of TER, with many independent studies showing an interaction between sodium and chloride transport mediated by CFTR [32–34]. The combined data suggest that ion transport at baseline and after sequential exposure to drugs both vary but amiloride-sensitivity as a marker of the underlying sodium transport remains the major component. In fact amiloride reduced by 10-fold the reference range for the current transported by the epithelium (data not shown) and, as might be expected, widens the TER range (Fig 4B and 4C). Importantly the order of drug administration does not alter the width of the TER range (compare Fig 4C-I with 4C-II and S2 Fig). Only 4 subjects have low values of TER after amiloride (B,D,E and G, below 1000 Ω·cm^2^, S4 Fig, panels B-I and B-II) but only two of them (E and G) have little change in TER after its administration (see S6 Fig, TER Ratio near to 1).

From a disease perspective, the magnitude of TER has been reported to be important in the pathogenesis of asthma [2] and future work will have to determine whether the starting values of TER might determine the propensity of a given individual to develop clinically detectable disease, for example after exposure to diesel particulates. Mechanistically, recent work has shown that a number of genes alter the phenotype of Cystic Fibrosis [35,36] and some of these are epithelial transporters whereas others are immune modulators. Co-inheritance of differences in such proteins are equally likely to be present in our normal volunteers and might explain the background variability in the parameters reported in this paper. This idea is supported by the finding that magnitude of the forskolin current in the presence of amiloride, and its subsequent level after the addition of the CFTR inhibitor, is also variable (data not shown). Between donors, this means that the airway epithelium is reacting differently to the presence of different drug combinations and care is needed in the choice of control subjects in a disease setting.

We believe these are important considerations for personalized medicine, particularly when choosing the best controls for a study.

We report a dichotomous TER response that generates two drug-discriminative populations that are only revealed post amiloride. This group difference is further exemplified as different profiles when the TER responses are normalised to either an initial baseline value, or re-calculated at each drug addition step on a rolling baseline basis: both approaches are needed in order to identify outliers, reflective of extreme response to a given drug or to drug combinations. An example is given in S6 Fig with 99% confidence intervals shown in the shaded areas; the outliers are clearly shown irrespective of whether the initial (panels A-I, A-II) or the rolling baseline (panels B-I,B-II) is chosen as the starting value when calculating the ratio of changes.

Currently funded to study a rare inherited airway disease, Birt-Hogg-Dubé Syndrome [37], we faced the problem of limited access to airway tissue. Therefore we focused on studying apparently healthy volunteers to define a reference range of electrophysiological parameters, a mandatory analysis prior to asking patients to volunteer for airway research studies [38–40].

In pilot work we tested the utility of our SOP in brushings from donors affected by different diseases: three asthmatics, one BHD patient and four uninfected CF patients (homozygotes F508del-CFTR). For the asthmatics and BHD patients we were able to expand, differentiate (with an average of about twenty reconstituted epithelia for each donor) and analyse the electrophysiological parameters of the resultant ALI cultures (data not shown). To our surprise, none of the CF patient cells would attach and expand like either the controls or the disease-affected subjects. Further work, outside the scope of the current paper, will have to establish the cause (which was not due to bacterial or yeast infection).

By way of possible explanation, we note that over recent decades, many workers in the CF field [41] report abnormalities in cellular networks that might explain such very unusual growth characteristics in our CF brushings, consistent with the many independent pathways that are abnormal after F508 is deleted from the CFTR protein [42,43]. For example, some of these pathways centre on protein that binds to the first nucleotide binding domain of CFTR and controls the signalling flux between the epidermal growth factor receptor and the assembly of the scaffold which approximates MEK with ERK in the process of cell growth and differentiation.

Thus our SOP could in future help determine the means to repair the abnormal pathways that alter the production of epithelia from nasal brushings.

To this end, we present our range of values from our SOP that might help others navigate the data obtained by different groups who often take polar opposite positions on which ion transport characteristics are the principal drivers of pathophysiological change in a given disease. Importantly, we observe that a given individual can generate a mean/median *in vitro* response that is reflective of their own set of ALIs as a whole, with the caveat that sufficient numbers of cells must be harvested/cultured per nasal biopsy to permit sufficient ALIs to be generated to compensate for seeding progenitor variability. We propose that a minimum of 5–6 ALIs per donor are necessary when performing ion transport experiments for better interpretation and hope that our data will help different groups faced with the challenges of understanding the nature of the ‘normality challenge’ in their chosen disease. That challenge has recently been set out in a review of the issues researchers will have to overcome to compensate for the variability in the background on which disease occurs, which is the holy grail of personalised or precision medicine [44].

## Supporting Information

S1 Fig Effect of Na+ depletion on the electrophysiological properties of human airway epithelia. (A) Recorded voltage from two different Ussing chamber experiments (donors C and K) in which the epithelia were exposed to forskolin (FSK, 1μM) and then 50% of the buffer was exchanged apically (white arrows) with NMDG^+^-HBSS (NaCl replaced with (NMDG^+^)Cl, NaHCO_3_ with KHCO_3_ and Na_2_HPO_4_ with KH_2_PO_4_). For volume compensation the basolateral side had the same 50% volume exchange but with standard HBSS. After stabilization, NaCl was added back by exchanging 50% of the buffer with standard HBSS (137.93 mM NaCl) for three times (grey arrows). (B) Changes in V, TER and I_Eq_ at baseline (BAS.), after the addition of forskolin (FSK) and at the different concentration of Na^+^ achieved after replacing each time 50% of the buffer in the apical chamber with the same volume of NMDG^+^-HBSS until 2.2mM NaCl was reached.
(TIFF)

S2 Fig Frequency distribution of TER values for all of the ALIs at baseline and after manipulation of ion transport. (A) Relative frequency distribution of TER values at baseline for all of the 137 ALIs analysed. The X-axes indicates the center of the bins in which the range of all TER values have been divided. Each bin has a width of 200 Ω·cm^2^, meaning that the range of each bin is “Bin center ± 100”. The Y-axes indicates the relative frequency (%) of ALIs that fall into each bin. Relative frequency distribution at baseline and after the addition of drugs (B-I) for the 44 ALIs analysed with drug regime I and (B-II) for the 81 ALIs analysed with drug regime II.
(TIFF)

S3 Fig Baseline values analysis at donor level. Mean baseline electrophysiological values of (A) voltage, (B) resistance (TER) and (C) calculated equivalent current (V/TER[I_Eq_]) from 18 donors (A-R) ranked in ascending order with respect to mean TER value. The data show normal distribution (Shapiro-Wilk test, P>0.05) for V and TER but not for V/TER[I_Eq_] (P<0.001).
(TIFF)

S4 Fig Analysis of TER values upon drug addition. Before-after graphs of TER values from (A-I) drug regime I and (A-II) drug regime II, showing the changes in TER for all of the individual ALIs after each sequential drug addition. Mean TER values per donor (A-R, ranked in alphabetic order as derived from Fig 3B in the manuscript) in (B-I) drug regime I and (B-II) drug regime II. Data are presented as means ± SEM.
(TIFF)

S5 Fig ALI-based analysis of TER ratio change upon drug addition. (A-I, A-II) Ratio of TER change with drug regime I (n = 44) and II (n = 81) respectively, applying initial baseline approach highlighting the cumulative effect of the drugs. (B-I, B-II) Same data analysed applying the rolling baseline approach to the TER values of individual ALIs. Error bars: mean ± 95% CI.
(TIFF)

S6 Fig Analysis of TER ratio at donor level (A-R) for the two drug regimes. (A-I, A-II) Mean TER ratio in drug regime I (N = 13) and II (N = 18) respectively; donors ranked in ascending order with respect to amiloride-induced change in TER (+AMI/BAS.), applying the initial baseline approach. (B-I, B-II) Same as above with donors ranked in ascending order with respect to amiloride-induced change in TER (+AMI/Inh172 in B-I and +AMI/BAS in B-II.), applying the rolling baseline approach. Data shown as means ±SEM; Boxes: mean ±99%CI for all ALIs.
(TIFF)

S7 Fig Relationship between baseline voltage and change in TER after amiloride addition. Baseline V plotted against ratio of TER upon amiloride addition in drug regime I (A-I, C-I), and drug regime II (A-II). Second order polynomial regression was performed; line of regression with 99%CI (dotted lines). (B-I, D-I, B-II) Logarithm of baseline V plotted against TER ratio with amiloride.
(TIFF)

S1 File Additional Methods: Study Approval, Cell culture additional considerations, Immunofluorescence and Trans-epithelial resistance (TER). Table A. Mean baseline electrophysiological values from Ussing chamber experiments of individual donors. Table B. Regression and statistical analysis of Fig 5. Table C. Regression and statistical analysis of Fig 6. Table D. Regression and statistical analysis of Fig 8. Table E. Mean TER values for Drug regime I. Table F. Mean TER values for Drug regime II. Supporting Information References.
(DOCX)
