# Supplementary figures and images for: Using Drugs to Probe the Variability of Trans-Epithelial Airway Resistance
Source: PLoS One. 2016 Feb 29;11(2):e0149550. doi: 10.1371/journal.pone.0149550 (PMC4771809; doi:10.1371/journal.pone.0149550)

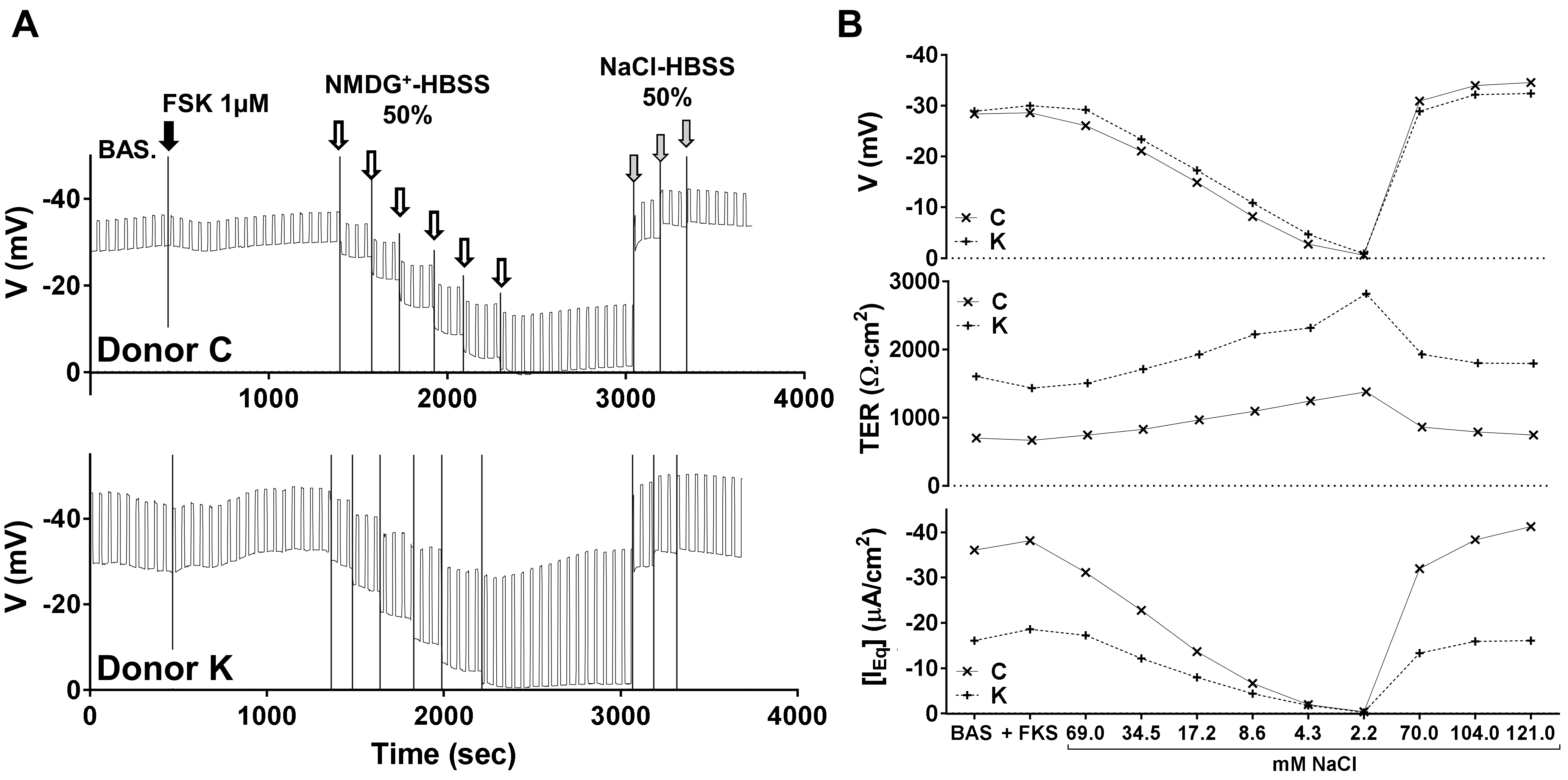

Supplement: S1 Fig — (A) Recorded voltage from two different Ussing chamber experiments (donors C and K) in which the epithelia were exposed to forskolin (FSK, 1μM) and then 50% of the buffer was exchanged apically (white arrows) with NMDG+-HBSS (NaCl replaced with (NMDG+)Cl, NaHCO3 with KHCO3 and Na2HPO4 with KH2PO4). For volume compensation the basolateral side had the same 50% volume exchange but with standard HBSS. After stabilization, NaCl was added back by exchanging 50% of the buffer with standard HBSS (137.93 mM NaCl) for three times (grey arrows). (B) Changes in V, TER and IEq at baseline (BAS.), after the addition of forskolin (FSK) and at the different concentration of Na+ achieved after replacing each time 50% of the buffer in the apical chamber with the same volume of NMDG+-HBSS until 2.2mM NaCl was reached. (TIFF) [file pone.0149550.s001.tiff]

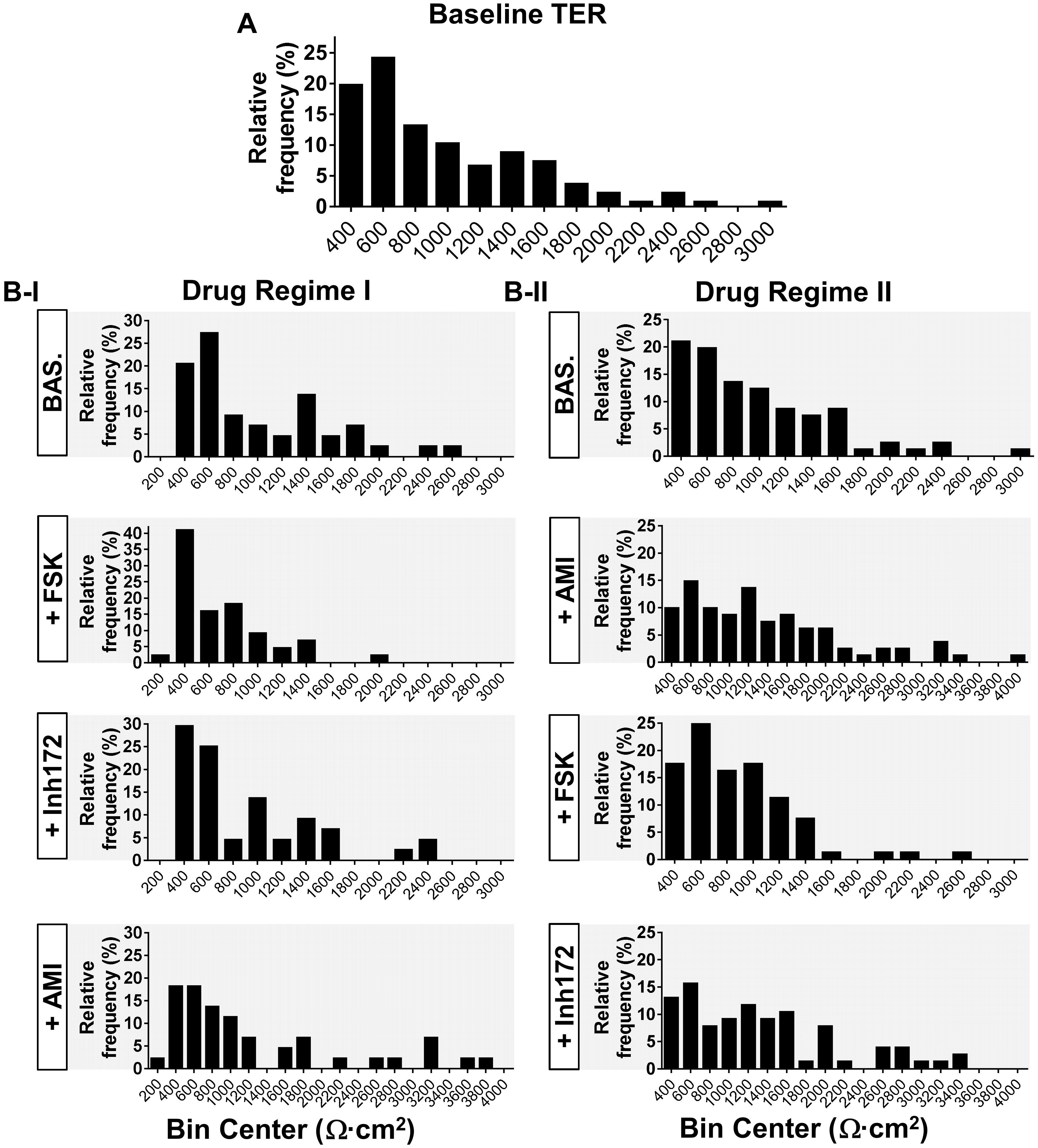

Supplement: S2 Fig — (A) Relative frequency distribution of TER values at baseline for all of the 137 ALIs analysed. The X-axes indicates the center of the bins in which the range of all TER values have been divided. Each bin has a width of 200 Ω·cm2, meaning that the range of each bin is “Bin center ± 100”. The Y-axes indicates the relative frequency (%) of ALIs that fall into each bin. Relative frequency distribution at baseline and after the addition of drugs (B-I) for the 44 ALIs analysed with drug regime I and (B-II) for the 81 ALIs analysed with drug regime II. (TIFF) [file pone.0149550.s002.tiff]

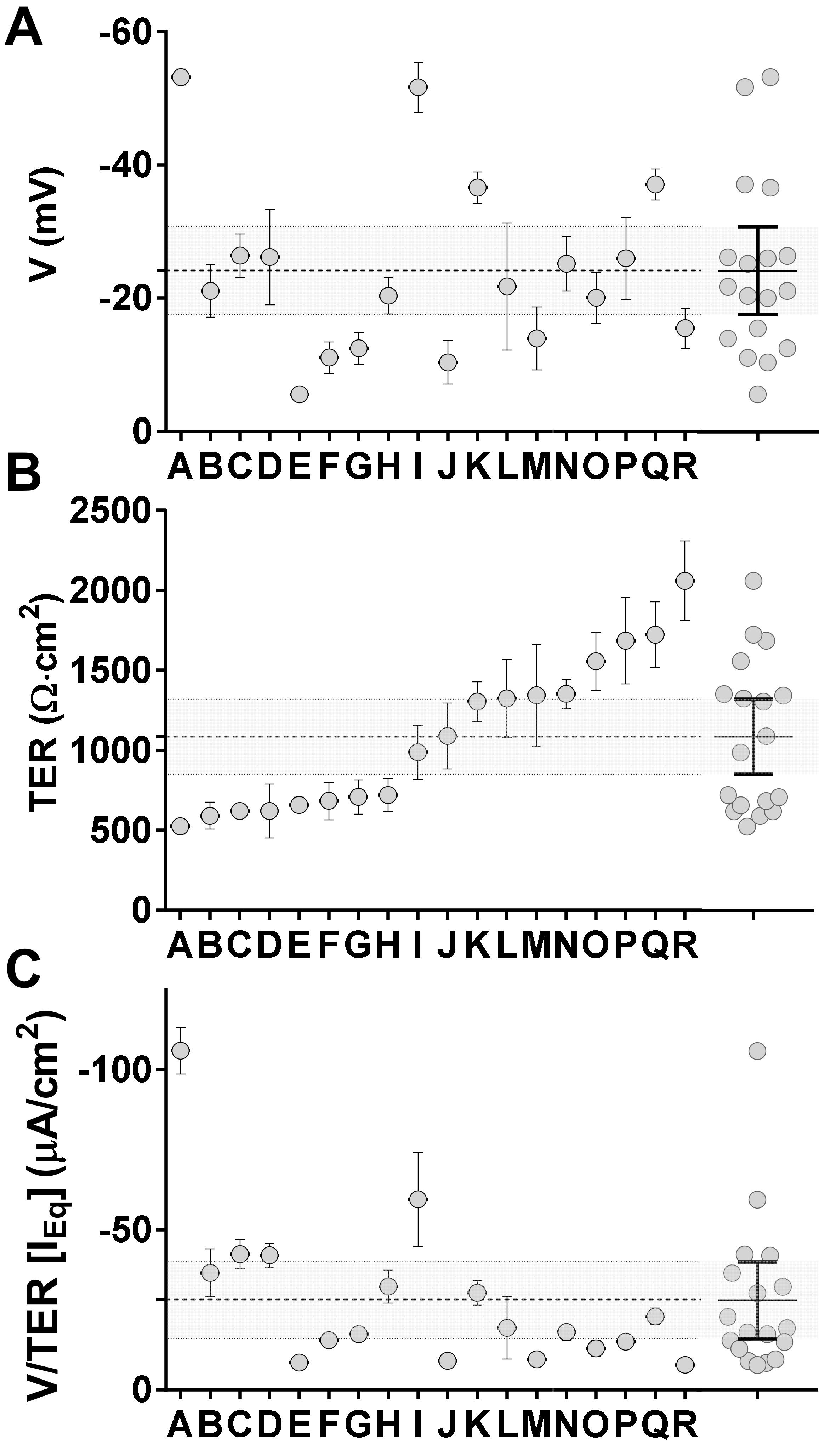

Supplement: S3 Fig — Mean baseline electrophysiological values of (A) voltage, (B) resistance (TER) and (C) calculated equivalent current (V/TER[IEq]) from 18 donors (A-R) ranked in ascending order with respect to mean TER value. The data show normal distribution (Shapiro-Wilk test, P>0.05) for V and TER but not for V/TER[IEq] (P<0.001). (TIFF) [file pone.0149550.s003.tiff]

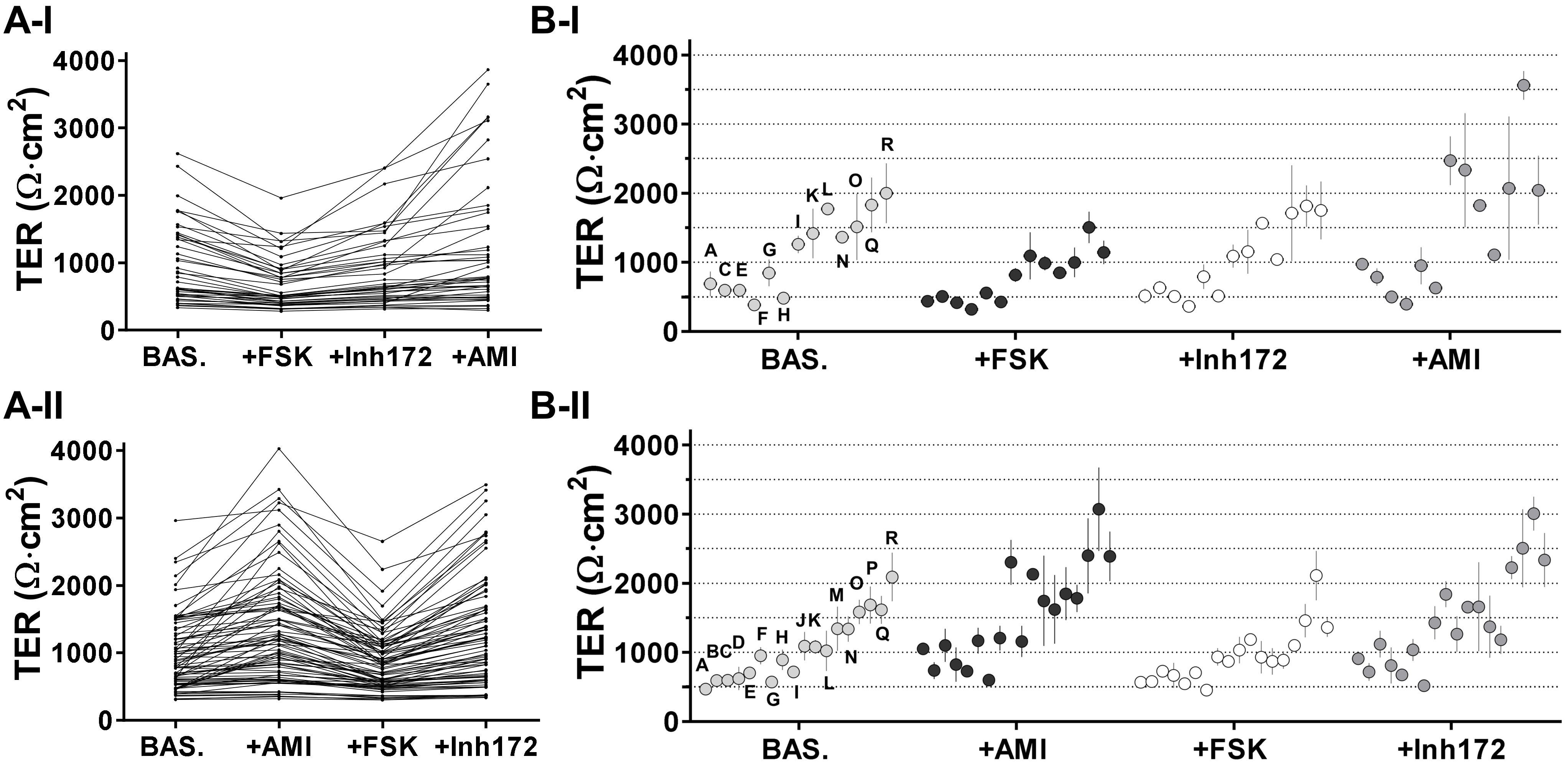

Supplement: S4 Fig — Before-after graphs of TER values from (A-I) drug regime I and (A-II) drug regime II, showing the changes in TER for all of the individual ALIs after each sequential drug addition. Mean TER values per donor (A-R, ranked in alphabetic order as derived from Fig 3B in the manuscript) in (B-I) drug regime I and (B-II) drug regime II. Data are presented as means ± SEM. (TIFF) [file pone.0149550.s004.tiff]

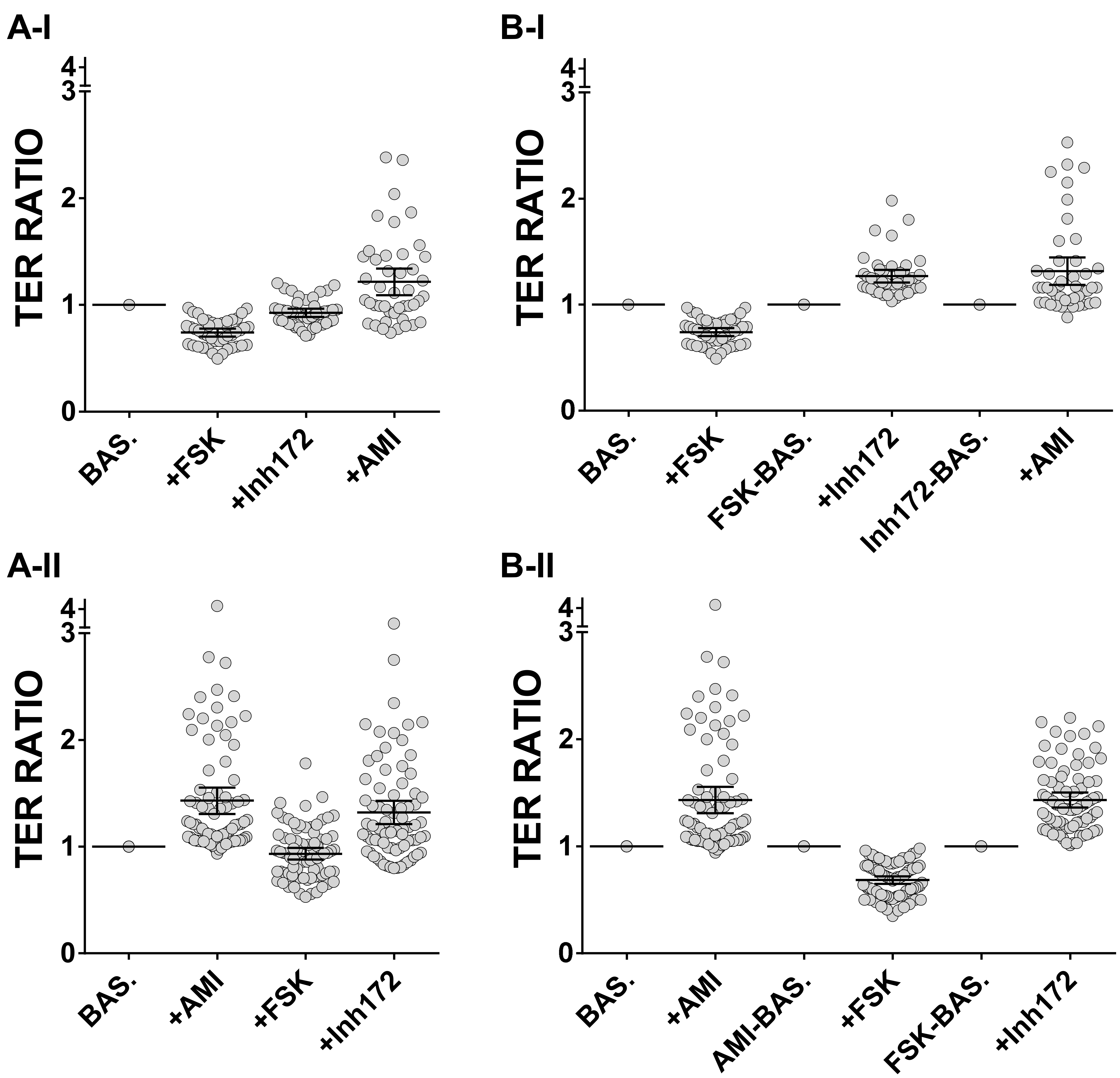

Supplement: S5 Fig — (A-I, A-II) Ratio of TER change with drug regime I (n = 44) and II (n = 81) respectively, applying initial baseline approach highlighting the cumulative effect of the drugs. (B-I, B-II) Same data analysed applying the rolling baseline approach to the TER values of individual ALIs. Error bars: mean ± 95% CI. (TIFF) [file pone.0149550.s005.tiff]

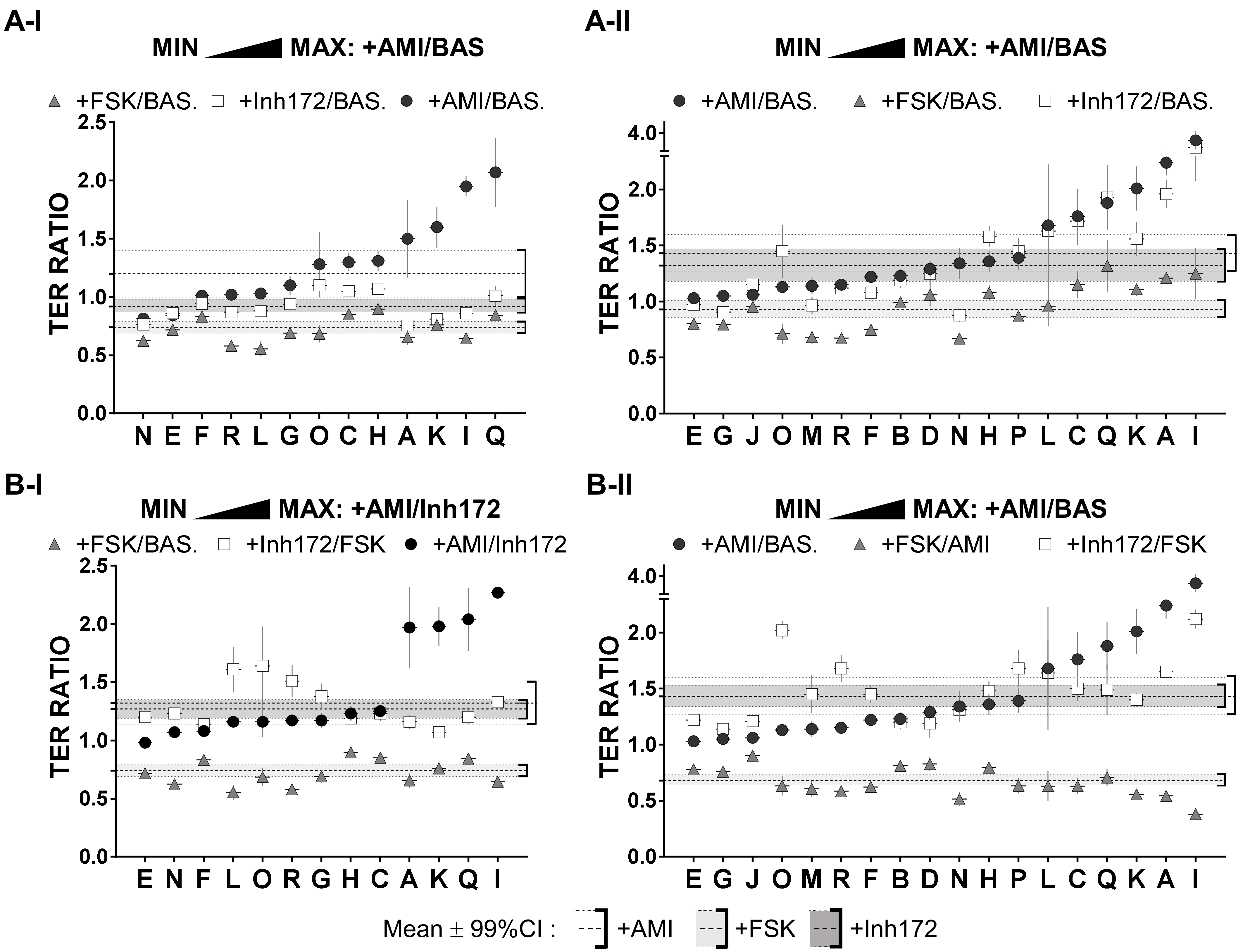

Supplement: S6 Fig — (A-I, A-II) Mean TER ratio in drug regime I (N = 13) and II (N = 18) respectively; donors ranked in ascending order with respect to amiloride-induced change in TER (+AMI/BAS.), applying the initial baseline approach. (B-I, B-II) Same as above with donors ranked in ascending order with respect to amiloride-induced change in TER (+AMI/Inh172 in B-I and +AMI/BAS in B-II.), applying the rolling baseline approach. Data shown as means ±SEM; Boxes: mean ±99%CI for all ALIs. (TIFF) [file pone.0149550.s006.tiff]

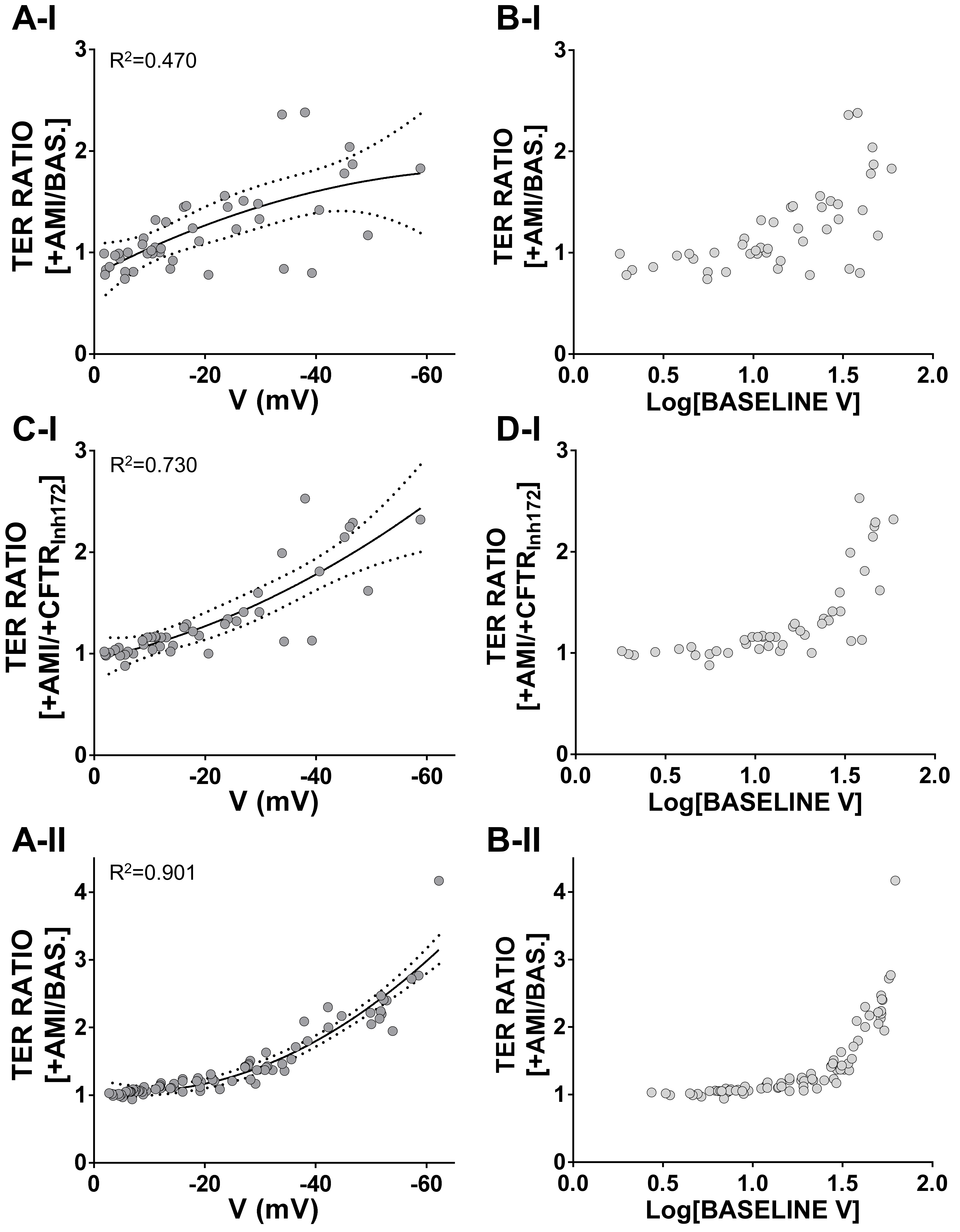

Supplement: S7 Fig — Baseline V plotted against ratio of TER upon amiloride addition in drug regime I (A-I, C-I), and drug regime II (A-II). Second order polynomial regression was performed; line of regression with 99%CI (dotted lines). (B-I, D-I, B-II) Logarithm of baseline V plotted against TER ratio with amiloride. (TIFF) [file pone.0149550.s007.tiff]
