# Supplementary material for: Using Drugs to Probe the Variability of Trans-Epithelial Airway Resistance
Source: PLoS One. 2016 Feb 29;11(2):e0149550. doi: 10.1371/journal.pone.0149550 (PMC4771809; doi:10.1371/journal.pone.0149550)
Supplement: S1 File — Table A. Mean baseline electrophysiological values from Ussing chamber experiments of individual donors. Table B. Regression and statistical analysis of Fig 5. Table C. Regression and statistical analysis of Fig 6. Table D. Regression and statistical analysis of Fig 8. Table E. Mean TER values for Drug regime I. Table F. Mean TER values for Drug regime II. Supporting Information References. (DOCX) [file pone.0149550.s008.docx]

**Using drugs to probe the variability of transepithelial airway resistance**

Kendra Tosoni, Diane Cassidy, Barry Kerr, Stephen C. Land, Anil Mehta.

**SUPPORTING INFORMATION**

**Study Approval**

The brushing procedure was approved by East of Scotland Research Ethics Service (EoSRES) REC1 (REC reference 12/ES/0081 Protocol number 2011RC20) whereby the donor provides the brushing *pro bono* and on a signed informed consent basis thus relinquishing cell ownership rights. The consents are written such that any brushed cells can be cultured and transported globally for use by academic groups or by industry with cost recovery. For third party use, upon receipt by the University of Dundee of an agreed material transfer agreement, this protocol ensures that any recipient institution only pays the technical cost of generating an ALI insert plus all transport costs. The consent procedures and documentation are available on request.

**Cell culture additional considerations**

Key findings:

(1) All media for cell harvest, proliferation and differentiation were of commercial grade to permit reproduction of the methods. These media generated monolayers of submerged cells on T25 flasks that contained mostly small triangular cells (Fig 1B in the manuscript).

(2) >95% volunteers generated P01 cells with approximately 50% of them also producing P03 cultures; P03 cultures alone were generated in only one volunteer.

(3) The pilot phase showed that submerged cells could be successfully frozen and revived after the P01 stage enabling banking for future studies (not shown). For the study reported here, no banked cells were used.

Notes:

1. Collagen stock solution is diluted in sterile PBS to give 5 µg/cm^2^ coverage and added to the surface to coat for at least 1 hour at 37°C. Incubation may be also carried out overnight at 4°C, warming dishes prior to use. It is very important to thoroughly rinse the coated surface with at least 2 changes of PBS prior to use.
2. From a donor’s brushing around 150 µl packed volume of cells per centrifuge tube was recovered, these are pooled and divided equally between 2xT25 flasks, larger pellets can be rescued onto 3xT25 flasks to increase cell recovery. Similarly when the pellet was found to be much smaller this was recovered into 1xT25. However we must point out that the size of cell pellet does not guarantee a good recovery and that patient variability affects this therefore we tend to stick with recovery onto 2xT25 flasks.
3. If the cells are still clumping after the first PBS wash it is advisable to repeat the washing process in order to get a more homogeneous cell suspension. If after the second wash the cells are still clumpy we pass them through a 19 and then a 21 Gauge needle to break down these clumps as required.
4. A faster spin (200 *g* to check) can be performed on the initial PBS used for cell wash: we have had success in growing patient fibroblast cultures using this method in approx. 50% of the patient samples we have tried. These cells were recovered in DMEM/F12 + 10% FBS + 100µg/ml Primocin, and took up to 4 weeks to show colony growth which we then expanded into cell lines that had approx. 8 to 10 passages before reaching senescence.
5. 80-90% confluent monolayers in T25 flasks are achieved on average after 7-10 days. Slowly growing HNE cultures should be used at whatever confluence they attain by no more than day 10.
6. Given the variability in the cell growth between donors, surface area conversion as a method of seeding was used to ensure production of confluent monolayers on the filters. When counting and seeding to a certain density, cell growth can vary greatly depending on the donor. We found that by seeding our snapwells/transwells at a higher seeding density ensured that we have a good fully covered support prior to shifting the cells to ALI. This equals, on a surface area comparison, to seeding each Snapwell/Transwell respectively with ~2.8 and ~1.3 times the amount of cells per cm^2^ with respect to the initial 80-90% confluent T25. With this seeding protocol, on average we recover 12 Snapwells and 2 Transwells from each P01 and P03 monolayers (2xT25 each). If lower confluence cultures are used, the seeding is proportionally reduced. Performing cell counting for seeding can be done, but monitoring the growth rate in the T25 is necessary as this can help decide cell seeding density. Our surface area conversion method equates to seeding approximately 1.5·10^5^ cell/cm^2^ when confluence was reached by day 8-10 while when confluence is reached earlier (day 6-7) a lower seeding density can be applied (a minimum of 8·10^4^ cell/cm^2^). When ALI is established, it is important to wash the apical surface of the epithelia at every fresh media change to ensure secretion and cell debris removal.
7. When culturing either Snapwells or Transwell, it is important to use only the proper recommended Corning 6 well dishes (Corning Costar, #3516) to ensure cell growth. Other manufactures 6 well dishes can accommodate them but a minimal reduction in depth of the wells can affect maintenance of cell viability.
8. Cells are cultured in 2X concentration of Pen/Strep and Primocin to ensure we have no issues regarding contamination especially when monitoring transepithelial electrical resistance with chopstick voltohmmeters regularly; this ensures maintenance of epithelia in culture for more than 2 months.
9. Figure 1a in the main text indicates a value of >400Ω on day 14 for airway resistance as threshold for subsequent analysis of the ALIs in Ussing chamber. This arbitrary limit was a pragmatic decision based on the finding that published data ([1,2], confirmed by our experience), show that there was no exact correspondence between the calculated resistance in the chamber and the EVOM2 measurements, in particular, the latter can lead to either under or over-estimation of the chamber values.

**Immunofluorescence**

Immunofluorescence was performed on HNE epithelia typically between 21 and 28 days, grown on either Transwell or Snapwells membranes, the latter fixed immediately after use in Ussing chamber. The membranes were washed 3 times with PBS (5 min each) then fixed by incubation in 4% Formaldehyde/PBS for 20 min at 4^o^C followed by 3 x 5 min further washes with PBS. The cells were permeabilised and any non-specific binding of primary antibody was prevented by incubation in PBS/0.3% Triton-X100 (PBST) containing 5% FBS for 1 hour at 4^o^C. Subsequent washes were all performed in PBST. Transwell membranes were divided into a maximum of 6 sections and Snapwell membranes were divided in half. Primary antibody incubation (monoclonal acetylated alpha tubulin and polyclonal Z0-1) at a 1 in 1000 dilution in PBST/1% BSA, was performed overnight at 4^o^C. The primary antibodies were removed by 3 x 5 min washes in PBST prior to incubation with secondary antibodies (Alexa Fluor 488 donkey anti-mouse IgG, and Alexa Flour 555 donkey anti-rabbit IgG) for 1 hour at 4^o^C in the dark, at a 1:2000 dilution in PBST/1%BSA. The sections were washed 3 times in PBST before being incubated for 5 minutes in PBST/DAPI (0.1 ng/µl) to visualize nuclei. After 3 further washes in PBST the membranes were mounted in Hydromount on glass microscopy slides. Images were collected using a Leica LSCM SP5 microscope, oil objective HCX PL APO. 63X/1.4, acquired with LAS AF and processed with Volocity.

**Trans-epithelial resistance (TER)**

The injection of pulses of current (I_inj_) resulted in deflections (delta-volts, ΔV) of spontaneous basal voltage (V) of that epithelium. The conventions and methods have been described elsewhere [19]. The magnitude of this deflection was used to calculate TER as the ratio of the measured change in joules of energy experienced by each coulomb of charge (i.e. voltage generated by the epithelium) divided by the experimenter applied pulse of injected current (TER=ΔV/I_inj_), multiplied by the surface area of the insert (corrected to 1 cm^2^). The equivalent short circuit current [I_Eq_] was calculated using Ohms law, I_Eq_=V/TER.

**Tables**

**Table A.** **Mean baseline electrophysiological values from Ussing chamber experiments of individual donors.**

| **Donor** | **BASELINE V**  **(mV)** | | **BASELINE TER**  **(Ω·cm^2^)** | | **BASELINE V/TER**  **[I_Eq_] (µA/cm^2^)** | | **Days at ALI** | | **ALIs** |
| --- | --- | --- | --- | --- | --- | --- | --- | --- | --- |
|  | **Mean** | **SD** | **Mean** | **SD** | **Mean** | **SD** | **Mean** | **SD** | **(n)** |
| **A** | -53.2 | 3.4 | 524.0 | 139.0 | -106.0 | 20.7 | 13.8 | 0.5 | 8 |
| **B** | -21.1 | 9.6 | 591.0 | 206.0 | -36.6 | 18.2 | 20.7 | 4.5 | 6 |
| **C** | -26.4 | 14.3 | 620.0 | 154.0 | -42.4 | 20.2 | 30.7 | 3.3 | 19 |
| **D** | -26.2 | 12.4 | 621.0 | 292.0 | -42.0 | 6.3 | 16.7 | 2.9 | 3 |
| **E** | -5.6 | 3.0 | 658.0 | 213.0 | -8.5 | 3.3 | 37.9 | 11.5 | 19 |
| **F** | -11.1 | 6.6 | 683.0 | 333.0 | -15.5 | 3.4 | 29.0 | 1.1 | 8 |
| **G** | -12.5 | 6.8 | 708.0 | 305.0 | -17.4 | 5.7 | 32.0 | 3.1 | 8 |
| **H** | -20.4 | 9.5 | 721.0 | 362.0 | -32.3 | 17.9 | 20.9 | 2.4 | 12 |
| **I** | -51.7 | 7.5 | 987.0 | 337.0 | -59.5 | 29.4 | 15.0 | 0.0 | 4 |
| **J** | -10.4 | 6.5 | 1089.0 | 413.0 | -9.1 | 2.6 | 34.0 | 0.0 | 4 |
| **K** | -36.6 | 6.6 | 1304.0 | 350.0 | -30.3 | 11.0 | 20.1 | 5.2 | 8 |
| **L** | -21.8 | 21.3 | 1324.0 | 545.0 | -19.4 | 21.8 | 31.4 | 5.4 | 5 |
| **M** | -14.0 | 10.6 | 1343.0 | 717.0 | -9.6 | 2.1 | 34.2 | 3.4 | 5 |
| **N** | -25.2 | 11.6 | 1352.0 | 252.0 | -18.0 | 7.2 | 39.0 | 2.0 | 8 |
| **O** | -20.1 | 8.6 | 1557.0 | 406.0 | -12.9 | 5.6 | 34.2 | 1.1 | 5 |
| **P** | -26.0 | 10.7 | 1686.0 | 467.0 | -15.0 | 2.7 | 33.0 | 0.0 | 3 |
| **Q** | -37.1 | 5.7 | 1723.0 | 503.0 | -22.9 | 6.4 | 22.0 | 3.2 | 6 |
| **R** | -15.5 | 7.4 | 2060.0 | 609.0 | -7.8 | 3.7 | 30.7 | 5.2 | 6 |

**Table B.** **Regression and statistical analysis of Fig 5.**

| **Fig** | **ALI (n)** | **AMI Responder** | **Equation** | **R^2^** | **Slope**  **t-test** | **Mann-Whitney U**  **Two-tailed** |
| --- | --- | --- | --- | --- | --- | --- |
| 5 A-I | 44 | - | Y= 0.597X + 99.7 | 0.888 | - | - |
| 5 B-I | 44 | - | Y= 0.904X + 8.90 | 0.930 | - | - |
| 5 C-I | 44 | - | Y= 1.42X -148 | 0.727 | - | - |
| **^1^** 5 D-I | 31 | LOW | Y= 0.979X + 6.38 | 0.926 | P<0.0001 | P=0.0006 |
|  | 13 | HIGH | Y= 1.68X + 115 | 0.853 |  |  |
| 5 A-II | 81 | - | Y= 1.26X + 141 | 0.689 | - | - |
| **^2^** 5 B-II | 57 | LOW | Y= 1.12X – 49.1 | 0.956 | P=0.0325 | P=0.0001 |
|  | 24 | HIGH | Y= 1.49X + 490 | 0.753 |  |  |
| 5 C-II | 56 | LOW | Y= 0.565X + 211 | 0.830 | P=0.0002 | P=0.0131 |
|  | 24 | HIGH | Y= 0.923X + 201 | 0.747 |  |  |
| 5 D-II | 56 | LOW | Y= 1.17X – 42.4 | 0.850 | P=0.345 | P=0.0003 |
|  | 21 | HIGH | Y= 1.34X + 465 | 0.680 |  |  |

**^1^** Grouping was decided based on whether the fold increase of TER after amiloride addition (+AMI/BAS. ratio) was above or below 1.42 (slope in 5 C-I).

**^2^** Grouping was decided based on whether the fold increase of TER after amiloride addition (+AMI/BAS. ratio) was above or below the mean ratio for the population (mean ratio=1.43).

**Table C.** **Regression and statistical analysis of Fig 6.**

| **Fig** | **ALI (n)** | **AMI Responder** | **Equation** | **R^2^** | **Slope**  **t-test** | **Mann-Whitney U**  **Two-tailed** |
| --- | --- | --- | --- | --- | --- | --- |
| 6 A-I | 44 | - | Y= 0.597X + 99.7 | 0.888 | - | - |
| 6 B-I | 44 | - | Y= 1.39X – 56.4 | 0.881 | - | - |
| 6 C-I | 44 | - | Y= 1.58X -168 | 0.788 | - | - |
| **^1^** 6 D-I | 35 | LOW | Y= 1.24X – 77.8 | 0.978 | P=0.0029 | P<0.0001 |
|  | 9 | HIGH | Y= 1.70X + 404 | 0.847 |  |  |
| **^2^** 6 A-II | 57 | LOW | Y= 1.12X – 49.1 | 0.956 | P=0.0325 | P=0.0001 |
|  | 24 | HIGH | Y= 1.49X + 490 | 0.753 |  |  |
| 6 B-II | 56 | LOW | Y= 0.453X + 250 | 0.819 | P=0.1293 | P=0.0131 |
|  | 24 | HIGH | Y= 0.544X + 42.7 | 0.762 |  |  |
| 6 C-II | 56 | LOW | Y= 1.95X – 386 | 0.906 | P=0.0002 | P=0.0003 |
|  | 21 | HIGH | Y= 1.35X + 276 | 0.796 |  |  |

**^1^** Grouping was decided based on whether the fold increase of TER after amiloride addition (+AMI/CFTR_Inh172_ ratio) was above or below 1.58 (slope in 6 C-I).

**^2^** Grouping was decided based on whether the fold increase of TER after amiloride addition (+AMI/BAS. ratio) was above or below the mean ratio for the population (mean ratio=1.43).

**Table D.** **Regression and statistical analysis of Fig 8.**

| **Fig** | **ALI (n)** | **AMI Responder** | **Equation** | **R^2^** | **Slope**  **t-test** | **Mann-Whitney U**  **Two-tailed** |
| --- | --- | --- | --- | --- | --- | --- |
| **^1^** 9 A-I | 31 | - | Y= 0.171X + 0.83 | 0.143 | - | - |
| **^2^** 9 B-I | 35 | - | Y= 0.256X + 0.87 | 0.491 | - | - |
| **^3^** 9 A-II | 57 | LOW | Y= 0.372X + 0.74 | 0.707 | P<0.0001 | P<0.0001 |
|  | 24 | HIGH | Y= 4.31X – 4.95 | 0.633 |  |  |

**^1^** Grouping was applied as in Fig 5 – Drug regime I.

**^2^** Grouping was applied as in Fig 6 – Drug regime I.

**^3^** Grouping was applied as in Figs 5 and 6 – Drug regime II.

**Table E.** **Mean TER values for Drug regime I.**

|  | **BAS.** | | | **+FSK** | | | **+Inh172** | | | **+AMI** | | |
| --- | --- | --- | --- | --- | --- | --- | --- | --- | --- | --- | --- | --- |
|  | **Mean** | **SD** | **ALI**  **(n)** | **Mean** | **SD** | **ALI**  **(n)** | **Mean** | **SD** | **ALI**  **(n)** | **Mean** | **SD** | **ALI**  **(n)** |
| **A** | 688 | 250 | 2 | 440 | 102 | 2 | 513 | 154 | 2 | 974 | 51 | 2 |
| **C** | 596 | 165 | 5 | 506 | 130 | 5 | 632 | 222 | 5 | 786 | 285 | 5 |
| **E** | 597 | 185 | 8 | 416 | 79 | 8 | 507 | 136 | 8 | 499 | 142 | 8 |
| **F** | 387 | 68 | 3 | 320 | 41 | 3 | 366 | 67 | 3 | 395 | 93 | 3 |
| **G** | 845 | 389 | 4 | 556 | 147 | 4 | 791 | 355 | 4 | 950 | 538 | 4 |
| **H** | 481 | 82 | 5 | 429 | 64 | 5 | 515 | 111 | 5 | 628 | 123 | 5 |
| **I** | 1259 | 177 | 2 | 816 | 140 | 2 | 1090 | 235 | 2 | 2470 | 500 | 2 |
| **K** | 1418 | 507 | 2 | 1094 | 482 | 2 | 1153 | 450 | 2 | 2333 | 1164 | 2 |
| **L** | 1774 | 19 | 2 | 988 | 146 | 2 | 1565 | 38 | 2 | 1822 | 43 | 2 |
| **N** | 1364 | 91 | 4 | 847 | 48 | 4 | 1040 | 66 | 4 | 1109 | 62 | 4 |
| **O** | 1513 | 681 | 2 | 999 | 305 | 2 | 1711 | 979 | 2 | 2073 | 1466 | 2 |
| **Q** | 1831 | 689 | 3 | 1507 | 394 | 3 | 1815 | 520 | 3 | 3560 | 359 | 3 |
| **R** | 1999 | 613 | 2 | 1142 | 242 | 2 | 1751 | 592 | 2 | 2043 | 705 | 2 |

**Table F.** **Mean TER values for Drug regime II.**

|  | **BAS.** | | | **+AMI** | | | **+FSK** | | | **+Inh172** | | |
| --- | --- | --- | --- | --- | --- | --- | --- | --- | --- | --- | --- | --- |
|  | **Mean** | **SD** | **ALI**  **(n)** | **Mean** | **SD** | **ALI**  **(n)** | **Mean** | **SD** | **ALI**  **(n)** | **Mean** | **SD** | **ALI**  **(n)** |
| **A** | 469 | 11 | 6 | 1050 | 115 | 6 | 566 | 37 | 6 | 909 | 79 | 3 |
| **B** | 591 | 206 | 6 | 737 | 299 | 6 | 578 | 178 | 6 | 717 | 314 | 6 |
| **C** | 594 | 176 | 6 | 1102 | 589 | 6 | 726 | 204 | 5 | 1119 | 433 | 5 |
| **D** | 621 | 292 | 3 | 824 | 432 | 3 | 665 | 314 | 3 | 811 | 454 | 3 |
| **E** | 703 | 230 | 11 | 726 | 247 | 11 | 546 | 137 | 11 | 674 | 209 | 11 |
| **F** | 951 | 250 | 4 | 1170 | 370 | 4 | 705 | 161 | 4 | 1035 | 320 | 4 |
| **G** | 571 | 129 | 4 | 599 | 154 | 4 | 452 | 94 | 4 | 516 | 132 | 4 |
| **H** | 892 | 391 | 7 | 1206 | 476 | 7 | 934 | 335 | 7 | 1429 | 633 | 7 |
| **I** | 716 | 121 | 2 | 2302 | 457 | 2 | 869 | 76 | 2 | 1842 | 265 | 2 |
| **J** | 1089 | 413 | 4 | 1158 | 452 | 4 | 1033 | 385 | 4 | 1266 | 513 | 4 |
| **K** | 1080 | 174 | 3 | 2128 | 109 | 3 | 1186 | 98 | 3 | 1655 | 53 | 3 |
| **L** | 1023 | 507 | 3 | 1746 | 1133 | 3 | 930 | 405 | 3 | 1659 | 1112 | 3 |
| **M** | 1343 | 717 | 5 | 1621 | 1110 | 5 | 871 | 428 | 5 | 1372 | 1008 | 5 |
| **N** | 1339 | 373 | 4 | 1848 | 764 | 4 | 888 | 253 | 4 | 1181 | 407 | 4 |
| **O** | 1586 | 307 | 3 | 1783 | 342 | 3 | 1102 | 91 | 3 | 2225 | 288 | 3 |
| **P** | 1686 | 467 | 3 | 2398 | 941 | 3 | 1461 | 419 | 3 | 2507 | 979 | 3 |
| **Q** | 1615 | 351 | 3 | 3071 | 1046 | 3 | 2111 | 616 | 3 | 3007 | 422 | 3 |
| **R** | 2091 | 699 | 4 | 2388 | 721 | 4 | 1361 | 273 | 4 | 2336 | 777 | 4 |

**References**

1. Karp PH, Moninger TO, Weber SP, Nesselhauf TS, Launspach JL, Zabner J, et al. An in vitro model of differentiated human airway epithelia. Methods for establishing primary cultures. Methods Mol Biol Clifton NJ. 2002;188: 115–137. doi:10.1385/1-59259-185-X:115

2. Gondzik V, Awayda MS. Methods for stable recording of short-circuit current in a Na+-transporting epithelium. Am J Physiol - Cell Physiol. 2011;301: C162–C170. doi:10.1152/ajpcell.00459.2010

19. Mansley MK, Wilson SM. Effects of nominally selective inhibitors of the kinases PI3K, SGK1 and PKB on the insulin-dependent control of epithelial Na+ absorption. Br J Pharmacol. 2010;161: 571–588. doi:10.1111/j.1476-5381.2010.00898.x
